# Identifying indicator species in ecological habitats using Deep Optimal Feature Learning

**DOI:** 10.1371/journal.pone.0256782

**Published:** 2021-09-10

**Authors:** Yiting Tsai, Susan A. Baldwin, Bhushan Gopaluni

**Affiliations:** Department of Chemical and Biological Engineering, University of British Columbia, Vancouver, Canada; Ton Duc Thang University, VIET NAM

## Abstract

Much of the current research on supervised modelling is focused on maximizing outcome prediction accuracy. However, in engineering disciplines, an arguably more important goal is that of feature extraction, the identification of relevant features associated with the various outcomes. For instance, in microbial communities, the identification of keystone species can often lead to improved prediction of future behavioral shifts. This paper proposes a novel feature extractor based on Deep Learning, which is largely agnostic to underlying assumptions regarding the training data. Starting from a collection of microbial species abundance counts, the Deep Learning model first trains itself to classify the selected distinct habitats. It then identifies indicator species associated with the habitats. The results are then compared and contrasted with those obtained by traditional statistical techniques. The indicator species are similar when compared at top taxonomic levels such as *Domain* and *Phylum*, despite visible differences in lower levels such as *Class* and *Order*. More importantly, when our estimated indicators are used to predict final habitat labels using simpler models (such as Support Vector Machines and traditional Artificial Neural Networks), the prediction accuracy is improved. Overall, this study serves as a preliminary step that bridges modern, black-box Machine Learning models with traditional, domain expertise-rich techniques.

## Introduction

The main motivation of this work is to propose a Machine Learning-based feature extractor which works generally for non-linear datasets of high dimensionality, then assess its efficacy on a real biological case with pre-determined features, to determine whether it is sufficiently reliable to be used for other similar studies. Biologically-related disciplines are often faced with the task of modelling on high-dimensional datasets (i.e. many raw input variables). In disciplines such as disease diagnosis and prevention, much of the current research focus is on maximizing prediction accuracy of outcome labels. For example, the authors of [1] compared and contrasted the performances across a large arsenal of predictive models (ex. Logistic Regression, Support Vector Machines, Random Forests, Naives Bayes *k*-Nearest Neighbors) for the task of diabetes-2 diagnosis. However, in other disciplines such as ecology, dimensionality reduction and feature engineering are arguably equally important tasks, which aid the intuitive understanding of these datasets. In ecological datasets, community interactions between individual species (the input variables) can be difficult to model. Therefore, tasks such as the identification of *indicator species* (i.e. keystone or leader species) can be challenging, as the user is often perplexed as to which dimensionality reduction algorithm(s) are suitable. In past literature, methods such as *IndVal* [2] have traditionally been used for indicator species identification. Successful implementations can be seen in publications such as [3–5]), where abundance counts of microbial species are available from various sites. The *IndVal* method uses Multi-Dimensional Scaling [6] followed by Correspondence Analysis (CA) and Detrended Correspondence Analysis (DCA) [7] to rank the topology of sites. The indicator species groups are then identified using using Hierarchical [8] and *k*-means clustering [9]. The final indicator value of a particular species can be roughly described as its frequency of occurrence across all clusters, across all sites. However, due to the ever-increasing complexity and non-linear nature of modern datasets, the use of these traditional statistical tools may reduce the overall algorithm’s ability to generalize to a wide range of interactions between features.

Indicator species identification can be considered a special case of a more general category of modelling tasks, known as *feature analysis*. Given a data matrix ***X*** with *N* total samples as rows and *d* columns as raw variables, the precise definition of *feature analysis* can be subtly distinguished as the follows:

1. **Feature selection**: Determining whether each raw variable *x*_*j*_(*j* ∈ [1, ⋯, *d*]) contributes significantly to the model output(s).
2. **Feature extraction**: Determining linear or non-linear combinations of raw variables (ex. *x*_1_ ⋅ *log*(*x*_2_)) which contribute sigificantly to the model output(s).

The limitations of current state-of-art feature extraction methods are two-fold. The first problem is that many extractors simply use *feature selection* techniques, which are univariate in nature and are therefore unable to detect any multivariate interactions. More specifically, in some cases individual variables may not be relevant by themselves, but contribute significantly to the final outcome when they co-exist (in linear or non-linear fashions). The second problem is the limiting assumptions of the dimensionality reduction algorithms used in multivariate feature extractors. For example, extractors which prioritize the intuitiveness and interpretability of latent features often use Principal Component Analysis (PCA) [10] as a tool. This algorithm assumes that the underlying latent space is a linear combination of raw inputs (i.e. species), which may not be an accurate representation of many biological systems. Another example algorithm is *k*-means, which assumes that the underlying distributions of data are multivariate Gaussians (due to *k*-means being a special case of Gaussian mixtures [11]). If *k*-means were used to analyze a dataset which is highly non-Gaussian, then the results obtained could be misleading or meaningless.

In the current literature, most *feature selection* techniques are univariate approaches which assign “yes” or “no” labels to each raw variable, with respect to its relevance. A simple example is the addition of a *ℓ*_1_ or *lasso* regularizer [12] to any model objective function. Other methods include *Mean Decrease in Accuracy (MDA)* and *Mean Decrease in Gini (MDG)* [13], which are used in conjunction with Random Forest (RF) models. In MDA, individual features are permutated (scrambled) randomly and the resulting effect on model accuracy is determined, compared to the base-case where no features are scrambled. In MDG, the feature importance is determined by observing how many times a feature is used to create a decision split in the RF model. The disadvantages of these methods are mainly their univariate nature, as well as significant false discovery (positive or negative) rates.

On the other hand, *feature extraction* techniques attempt to recognize multivariate interactions between raw variables. A detailed review of the state-of-art methods in this area can be found in the work of [14]. One straightforward and computationally-inexpensive method is the aforementioned PCA, which discovers linear combinations of raw inputs. A previous publication of similar topic is [15], which used PCA to determine proteomic biomarkers. Another successful example is the work of [16], in which the authors used an enhanced PCA algorithm to correctly determine the latent features which best predict diabetic retinopathy in patients. In other cases, however, PCA may yield poor approximations due to the presence of non-linear latent features. These non-linearities can be partially mitigated by the use of methods such as Isometric Mapping (ISOMAP) [17], Locally Linear Embedding (LLE) [18], *t*-Stochastic Neighbor Embedding (*t*-SNE) [19], and Uniform Manifold Approximation and Projection [20]. The aforementioned algorithms provide lower-dimensional representations of data which are easy to visualize. For instance, a recent paper by [21] showed that *t*-SNE can be used to group similar eco-provinces in order to elucidate the community structures in each region. However, a major shortcoming of these non-linear methods is that extracted latent features are difficult to express in terms of the original raw variables. An example of this is the Supervised Locally Linear Embedding (SLLE) paper by [22]. In this work, the authors showed that three flower classes within the *Fisher Iris* dataset [23] can be compressed down to three distinct regions in a 2-***d*** space. However, the intuitive relationships between the 2 latent components and the original 4 features are never explicitly explored. Most multivariate dimensionality reduction methods prioritize either understanding of extracted features (ex. PCA), or easy visualization of clusters (ex. *t*-SNE), but few exist that satisfy both. Finally, the choice of dimensionality reduction method also depends on the amount of domain knowledge available. For example, when the functional structure of the latent features is well-known, then *kernels* [24] (a list of basis functions such as polynomials, square roots, exponentials, etc.) can be used to pre-determine the desired feature representation. On the other hand, if little to no domain knowledge is available, then the user resorts to fitting each kernel to find an appropriate one. If the non-linear structure is too complex, then none of the pre-established basis functions are suitable. Therefore, there exists a clear need for a dimensionality reduction procedure, which does not rely on copious amounts of prior knowledge.

Recent advances in computing power have supported the emergence of a powerful class of models known as Deep Learning (DL) [25]. These models are capable of training highly-accurate models to solve *classification* problems, due to deep neural networks being good *universal approximators* [26, 27] (i.e. capable of fitting any non-linearity using activation functions, if trained sufficiently). Works such as [28–31] demonstrate the high predictive capabilities of DL used in supervised learning cases. In these studies, the desired features are either pre-engineered using PCA, or by neural networks in *black-box* fashions that render them difficult to interpret. In applications that prioritize predictive accuracy, knowledge of the exact identities of the latent features may not be important. However, in other applications that prioritize diagnosis and root-cause analysis, the feature extraction step becomes critical in telling the user which combination variables to seek, and which to avoid. To the best of our knowledge, our work is one of few which explores the efficacy of DL models when designed to address both fronts: predictive accuracy and ability to identify the correct associated features.

In this work, we focus the application of this DL model to bioinformatics problems, an example being ecological habitat prediction and identification of indicator species associated with each habitat. These datasets include species abundance counts as samples; each sample consists of a large number of taxonomic units identified by 16*S* *r*RNA amplicon sequencing, represented either as Operational Taxonomic Units (OTUs) [32] or Amplicon Sequence Variants (ASVs) [33]. The corresponding outcome is usually a class label distinguishing its source (ex. lake, river, ocean, etc.) or its habitat (natural, disturbed, etc.). The goal of predictive modelling is to estimate the correct habitat label of new samples, given their abundance counts of all species. While the model’s test-set prediction accuracy is the focus of many ML research efforts, one can argue that remediation applications obtain more actionable information from feature extraction. Therefore, the motivation of our work in the context of bioinformatics applications can be clarified as follows:

1. Estimate the habitat label of each site using Machine Learning models, to produce a second set of labels which can be compared and contrasted to those obtained using traditional methods.
2. Identify the indicator species associated with each habitat, thereby eliminating the need to know abundance counts from all available species. This significantly reduces time and financial costs associated with manual sample collection and laboratory analysis.

We end this section by summarizing the scope, objectives, and advantages of our work as follows: develop a Deep Learning-based feature extractor for binary classification problems, with two main advantages over existing extractors:

1. It is agnostic to the true non-linear structure of the underlying feature space.
2. It still produces interpretable latent features.

We accomplish this by taking advantage of the universally-approximating property of Deep Learning, i.e. the ability of neural networks to learn any non-linear structure through brute-force combinations of activation functions (ex. *ReLU*, *SELU*, *sigmoid*, *tanh*, etc.). In order to maintain interpretability of the latent features, we use *ReLU* activations, which results in affine combinations of raw inputs, similar to those obtained from PCA. Finally, the general and case-specific objectives of our work can be summarized as follows:

1. Build and train a Deep Learning model for binary classification, which predicts the class labels of new samples with sufficiently high accuracy.
2. Identifies a latent space that optimally separates the two classes, which is then used to extract the most relevant features for prediction.
3. Demonstrate the efficacy of the proposed method on a real ecological dataset, by comparing the predicted class labels and extracted features with those identified previously using traditional methods.

In this paper, we will conform to standard Machine Learning notation regarding numerical matrices and vectors associated with datasets. The nomenclature will mirror those used in texts such as [25] and [11].

## Nomenclature

The following is a compiled list of abbreviations used within this manuscript:

ANN: Artificial Neural Net

ASV: Amplicon Sequence Variants

CA: Correspondence Analysis

DCA: Detrended Correspondence Analysis

DL: Deep Learning

GAN: Generative Adversarial Network

IID: Independent and Identically Distributed

*k*: Number of latent features

*k*^[*l*]^: Number of neurons in the *l*^*th*^ hidden layer of a DL model

ISOMAP: Isometric Mapping

LLE: Locally Linear Embedding

MA: Moving Average

MDA: Mean Decrease in Accuracy

MDG: Mean Decrease in Gini

MDS: Multi-Dimensional Scaling

ML: Machine Learning

*NaN*: Not a Number

OTUs: Operational Taxonomic Unit

PCA: Principal Component Analysis

PCoA: Principal Coordinate Analysis (equivalent to MDS)

*r*RNA: *r*-Ribosomal Ribonucleic Acid

RF: Random Forest

SVM: Support Vector Machine

*t*-SNE: *t*-Stochastic Neighbor Embedding

VAE: Variational Autoencoder

***x***^(*i*)^: The *i*^*th*^ data sample (all features)

***x***_*j*_: The *j*^*th*^ data feature (all samples)

$x_{j}^{\left(i\right)}$: The *i*^*th*^ sample of the *j*^*th*^ data feature

***z***^[*l*]^: The *l*^*th*^ hidden layer of a DL model

$z_{j}^{\left[l\right]}$: The *j*^*th*^ neuron in the *l*^*th*^ hidden layer of a DL model

## Materials and methods

**Fig 1:**
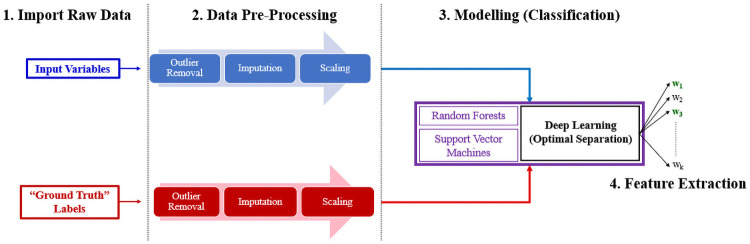
Overall data analysis workflow in block diagram form. *(Step 1)*: The collection of raw input data samples, as well as a corresponding set of labelled “ground-truth” targets. *(Step 2)*: The pre-processing of raw input data into suitable structures for modelling, guided by any available domain or expertise knowledge. *(Step 3)*: The training of several types of classification models (including Deep Learning), which maps inputs to their corresponding discrete class labels. *(Step 4)*: The design of special objective function within a Deep Learning classification, which identifies a latent space with improved class separation. The most dominant latent features are then distinguished by the magnitude (ex. *ℓ*_2_ norm) of the neural network weights.

The proposed modelling and feature extraction workflow will be performed on datasets of a general nature, by adhering to the workflow depicted in the following Fig 1:

The paper will provide a brief introduction of the methods employed for Steps 1 and 2. Most of the focus will be emphasized on Steps 3 and 4, which describe the concept behind the novel Deep Learning feature extractor as well as the interpretation of its results.

### Collection of raw data and ground truths

**Fig 2:**
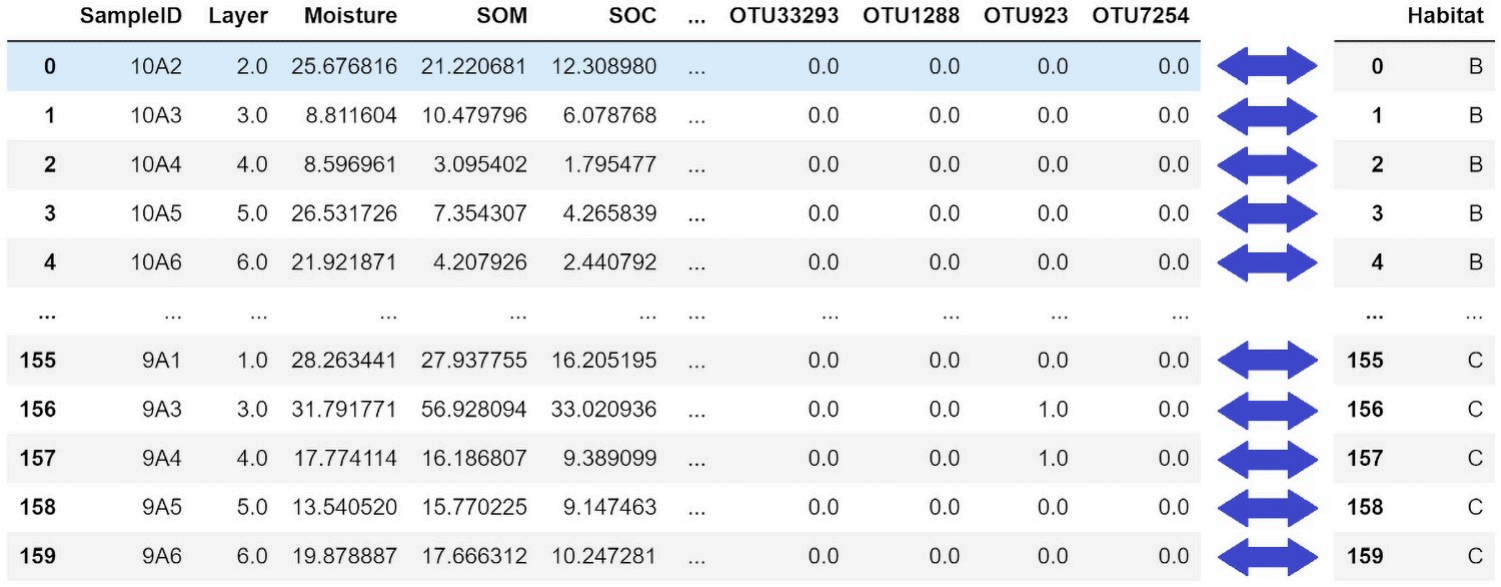
Printout of *pandas* dataframes containing raw data collected directly from ecological sites. *(Left)* Dataframe containing raw input variables. *(Right)* Dataframe containing output class labels.

Any modelling task requires the availability of data regarding the system of interest. An example of a combined chemical-biological dataset is shown in the following Fig 2.

In accordance with ML literature, the raw input data are structured in a $R^{N\times d}$ matrix that has *N* total rows representing the number data samples, and *d* total columns representing the number of raw variables. Inputs are usually continuous values; in some cases categorical labels may appear, but these can be discretized and transformed into numerical labels.

On the other hand, since this is a *classification* problem, the output or target variable is a set of discrete values. These represent the membership of each sample to a specific class, within a finite set of classes. Class labels can be easily transformed into numeric labels (ex. 0, 1, 2, etc.), even if they were not originally labelled as such. The accuracy of these outputs will inevitably affect the quality of any model trained using them. Therefore, the target variables for a dataset are often screened and scrutinized by domain experts and data scientists, before they can be ascertained as *ground truths*. They must be absolutely “correct” in the sense that few deviations from reality exist due to noise or other sources of error.

This work investigates the Mount Polley tailings storage breach, which occurred in the Quesnel region of British Columbia, Canada, in 2014. The paper [34] is one major publication which reports the disturbance to the soil and sediment microbiological communities (surrounding Mount Polley). The report is also accompanied by a dataset containing abundance counts of microbial species in every site; therefore, we will treat the findings and conclusions as “ground truths.” In other words, the binary habitat labels of each collected sample—*natural* or *disturbed*—were determined by soil ecology experts and are thus considered baselines for any new classification models. The following section provides more details on this case study as well as its implications.

#### Ground truth case study: Mount Polley tailings storage breach

In 2014, the Quesnel region (located in central British Columbia, Canada) suffered a major ecological disturbance, caused by the breach of the Mount Polley tailings dam. The released tailings material travelled and deposited into the nearby Hazeltine Creek, Quesnel, and Polley Lakes. The physical, biological, and ecological impacts of this breach have been thoroughly explored in publications such as [35] and [34], in attempt to characterize both the spatial and temporal changes within the microbial communities.

This paper will focus and expand upon the established results in [34], which has attempted to model the microbial community shifts using 16*S* *r*RNA and full metagenome sequencing. The main finding of [34] was that the identification of indicator species associated with natural and disturbed sites allowed the prediction of future biogeochemical trajectories in said sites. Specifically, ecologists could model just as accurately using the much smaller sub-set of biomarkers as when using the entire list of available species. This results in a significant reduction in both the complexity and cost of ongoing ecosystem health monitoring. Therefore, a strong justification can be made for the discovery of indicator species using other methods, such as Machine Learning, in order to complement those identified using the available statistical tools.

The data collected from the Mount Polley breach can be collected in a single input matrix ***X***, which contains *N* = 70 total samples as rows and *d* ≅ 22000 total raw variables as columns. Out of the 70 total samples, 41 belong to an undisturbed habitat, while the remaining 29 belong to a disturbed habitat. The exact descriptions of these *C* = 2 habitats (or classes) can be expanded as follows:

1. **Class 0**: Natural habitat of organic-rich soil related to a wetland community.
2. **Class 1**: Disturbed habitat originally consisting of native subtrate, which has been replaced by a mixture of fine tailings and sands.

The exact analysis steps of this study can be summarized as follows:

1. **Step 1**: Label each sample as either *natural* (Class 0) or *disturbed* (Class 1).
2. **Step 2**: Identify indicator species associated with either habitat.

**Fig 3:**
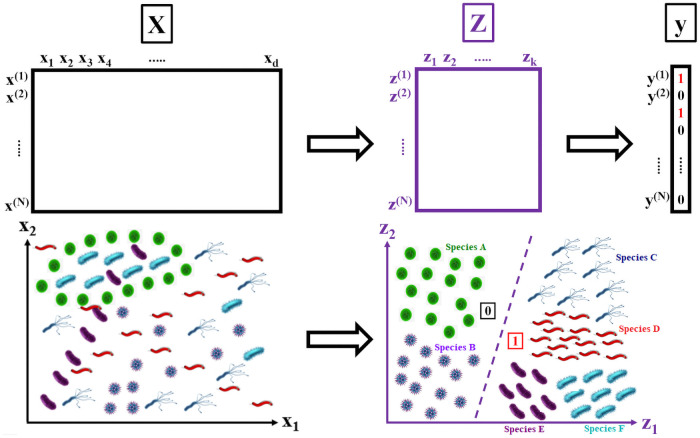
Discovery of an optimally-separating latent feature space. *(Top Left)* The high-dimensional and confounded raw inputs ***X*** makes class separation a challenging task.*(Bottom Left)* A case example including six microorganism species (A through F) which are entangled in the raw input space. *(Top Right)* A DL model which learns a latent space ***Z*** that optimally separates the classes. *(Bottom Right)* The disentanglement of the six species into distinct classes, which can be further aggregated into two major classes—Class 0 (A and B) and Class 1 (C through F).

The proposed feature extractor in this paper will be used to find an optimal latent space ***Z***, which serves as a transient between the mapping from ***X*** to the target habitats ***y***. Note that the raw input matrix ***X*** is considered “small-*N*-big-*d*” (i.e. few samples compared to a large number of raw variables), with a significant amount of noise confounding the raw variable space. Thus, traditional classification methods (ex. SVMs, RFs, feedforward networks, etc.) and clustering methods (ex. PCA, MDS, etc.) are expected to produce confounded class predictions or clusters with significant overlap. The desired transformation of the data space is illustrated in the following Fig 3.

### Pre-processing of biological variables

Due to the presence of microbial data in the form of species abundance counts, extra transformations were necessary to refine these entries. The abundance counts of many species are zero across most sites, thus resulting in a heavily low-skewing distribution. If these sparse counts are then used for modelling with no transformation, the models would place heavy emphasis on the many sparse species. This could result in spurious results caused by the vast negligence of highly-abundant but rarely-occurring species. In order to provide a more sensible count matrix which can be used for microbial community analysis, the following steps were taken:

1. **Removal of extremely sparse species**: The original count table includes 21721 total species. The abundance counts of each species was summed across all available samples. The counts of the top 50 species with respect to overall sum were selected as raw input variables.
2. **Log-transforms of remaining abundance counts**: In order to mitigate the low-leaning skew even more, a base-10 logarithmic transformation was applied to the remaining species counts, using the equation:
  $$\begin{array}{c} x_{species,log}=log_{10}\left(x_{species}+1\right) \end{array}$$ (1)
  The value of 1 was added at the end of Eq 1 to prevent taking logarithms of zero-counts.

**Fig 4:**
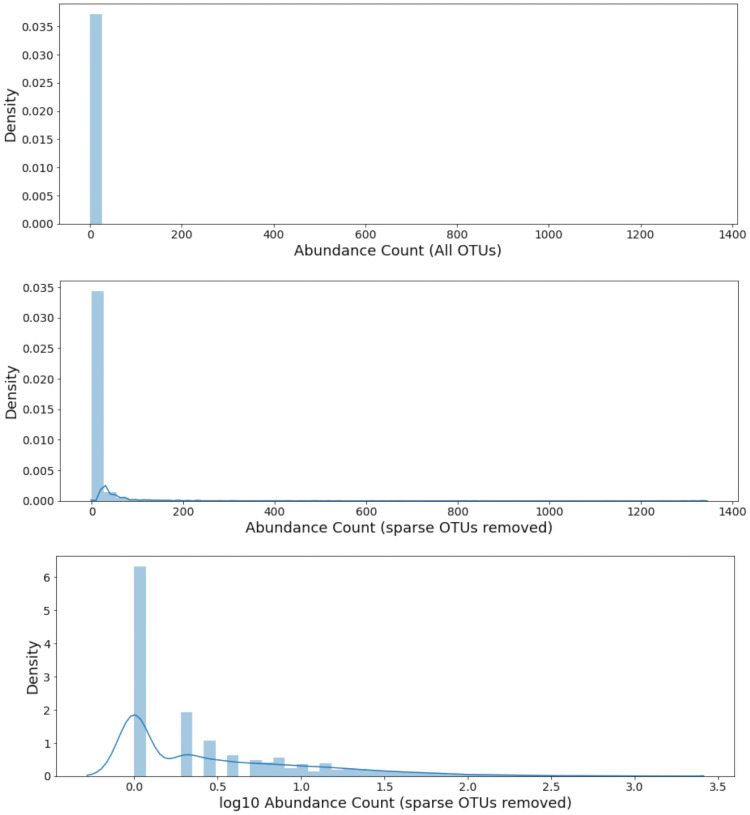
Species abundance counts after each pre-processing transformation. *(Top)* Distribution of counts from all 21721 species; the density is extremely skewed towards the low end. *(Middle)* Distribution of only the top 50 species by sum. *(Bottom)* Distribution of the top 50 species after a *log*_10_ transformation. Rarer but higher-abundance species are now recognizable.

**Fig 5:**
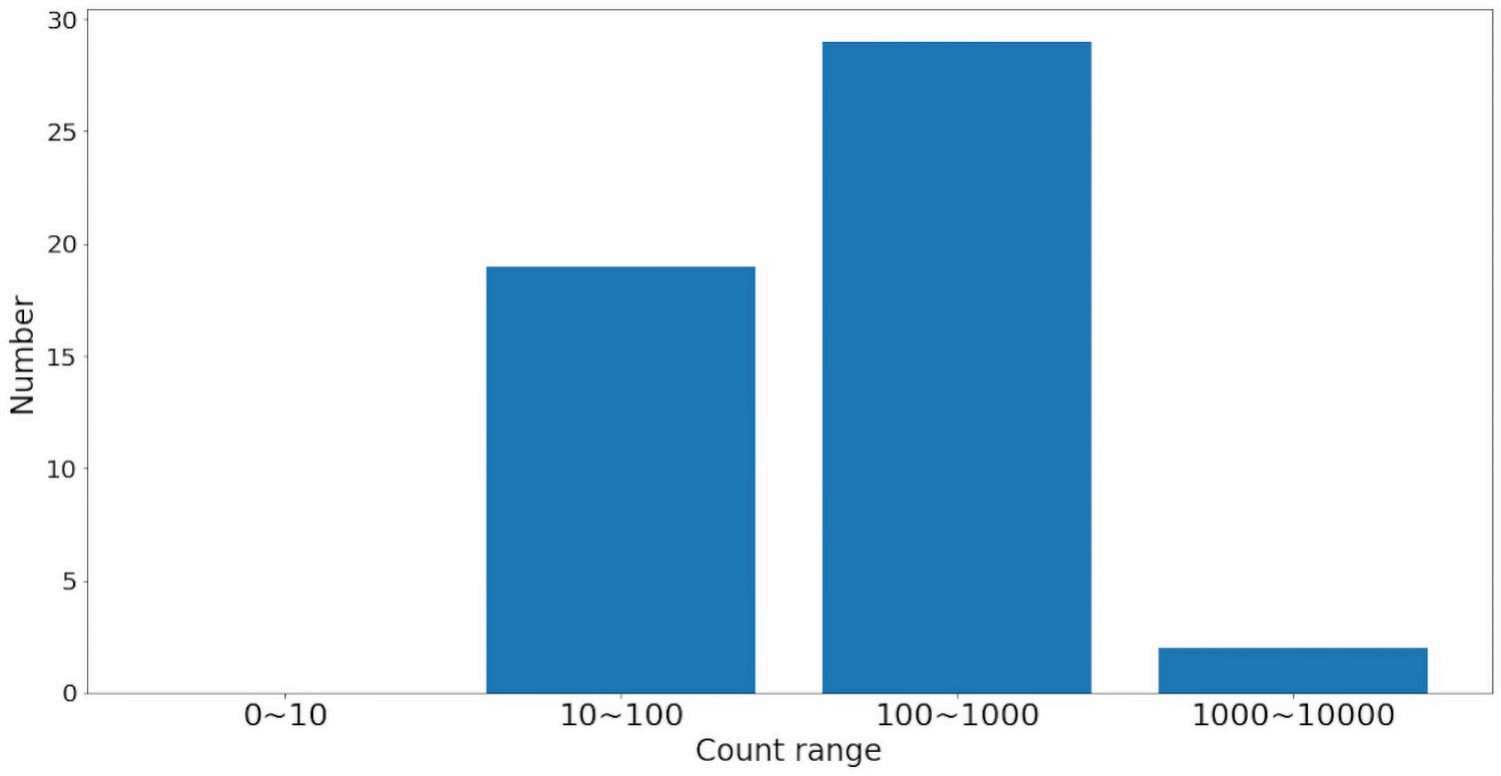
Histogram of maximum abundance counts.

The result of each subsequent pre-processing step can be observed in Fig 4, and the maximum abundance counts in each bracket are shown in Fig 5.

The roles of rarely-occurring species may be influential in a microbiological community, and ultimately decisive with regards to an outcome. In order to preserve the stark contrast in their high abundance compared to the many low-abundance species, an autoscaling transformation *was not applied* to the species counts. If one were applied, then each species would essentially have the same mean and variance in terms of counts, which would render them indistinguishable in subsequent modelling.

### Predictive modelling using machine learning

The case studies presented in this paper are categorized, in the ML field, as *classification* problems. A classification model describes a possible mapping between measured samples of raw, input variables ***X***, and corresponding measurements of a target variable ***y*** which are discrete membership (or class) labels. In contrast, a problem dealing with a continuous target ***y*** is known as a *regression* model. The methods presented in this paper will apply exclusively to *classification* and not *regression* problems.

The discussion will start with an introduction of two popular classification models—Random Forests (RFs) and Support Vector Machines (SVMs). However, these relatively simple models will only serve as a baseline of performance (i.e. model accuracy) to compare against. Most of the analysis will be focused on predictive classifiers using Deep Learning (DL) architectures, as well as its optimally-separating variant which we propose as an improved feature extractor.

### Traditional classification models

Two traditional classifiers are used in this work to obtain a baseline model performance, and they are:

1. Random Forests (RFs) [36]
2. Support Vector Machines (SVMs) [37]

RFs are a type of classifier in which the final membership label of each sample is decided by splitting each raw variable conditionally, in a binary manner. For example, one of the first splits in a RF model may be decided using the following set of binary conditions:

- ***x***_*j*_ ≥ thresh
- ***x***_*j*_ < thresh

Here, ***x***_*j*_ represents the *j*^*th*^ raw variable in the input data, and the threshold is a critical value which further decides how the data samples are classified. Since this process repeats throughout the *d* total available raw variables, the computational cost of exploring every possible combination becomes too astronomical to be feasible by conventional algorithms. RFs overcome this problem by splitting on a randomly-selected set of variables with each iteration, then *aggregating* across the results obtained from a large number of iterations. This strategy is known as an *ensemble* approach. It is often accompanied by *bootstrapping*, which selects a sub-sample of all available *N* data samples with replacement. Therefore, RFs are considered a member of the *bagging* (or *b*ootstrap *agg*regating) family of models.

**Fig 6:**
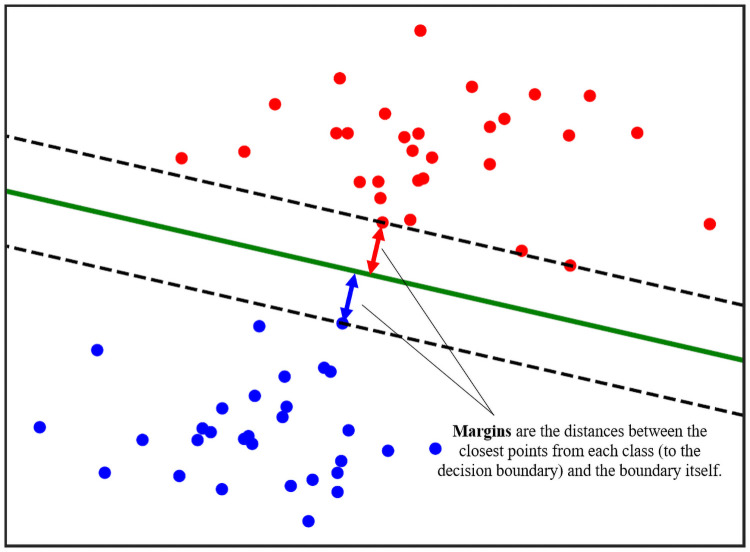
Visualization of a linear separating hyperplane and separating margins in a 2-class SVM model.

SVMs are a type of classifier which is popularly used in datasets with a modest number (i.e. tens) of raw variables *d* [11]. If *d* is too large, then SVM is susceptible to the *curse of dimensionality* phenomenon like many other ML models. In SVMs, the input data are assumed to take on a variable-space, where the different classes can be distinctly clarified by *separating margins*, which are illustrated in the following Fig 6.

In vanilla SVM, the *separating margin* is a linear hyperplane which is found by maximizing its respective distance to the closest members of each opposing class. Mathematically, this is achieved by minimizing the following Eq 2, known as the *hinge loss*:

$$\begin{array}{c} \underset{w}{min}\;\frac{1}{2}{\left|\left|w\right|\right|}_{2}\;+\;\sum_{i=1}^{N}\;max\left\{0,\;1-y^{\left(i\right)}w^{⊤}x^{\left(i\right)}\right\} \end{array}$$ (2)

The physical intuitions behind this loss can be interpreted, in words, as:

$$\begin{array}{c} \begin{array}{cc} \underset{\text{parameters}}{min}\;\;\;\;\; & \left\{\text{inverse}\;\text{of}\;\text{separating}\;\text{margins}\}\;\;+\;\;\{\text{misclassification}\;\text{rate}\right\} \end{array} \end{array}$$ (3)

Note that if the raw data do not conform to a linear structure, such as the example shown in Fig 6, then the vanilla SVM may fail to achieve high training and/or testing accuracy. These scenarios are traditionally tackled by use of the *kernel trick* [38]. This strategy transforms the raw input space into a higher-dimensional space, described by nonlinear basis functions which are determined *a priori*. If the non-linearity structure is well-known, then the kernel trick will efficiently transform the data into a linearly-separable form. Similarly, when the exact non-linear structure is unknown but the degree of non-linearity is low, then an adaptation of the kernel trick known as *Sparse Identification of Nonlinear Dynamics (SinDy)* [39] is equally powerful. However, in the worst-case scenario where both the structure and degree of non-linearity are high and unknown, there is no guarantee that a linearly-separable latent space can be found, even after a lengthy shopping-list of kernels has been exhausted.

This is where our proposed Deep Optimal Feature Learning comes into fruition. The following sections will outline how Deep Learning is generally used as classification models, as well as how we propose to shape the existing Deep Learning objective functions in order to achieve a latent space with improved class separation.

### The proposed deep optimal feature extractor

**Fig 7:**
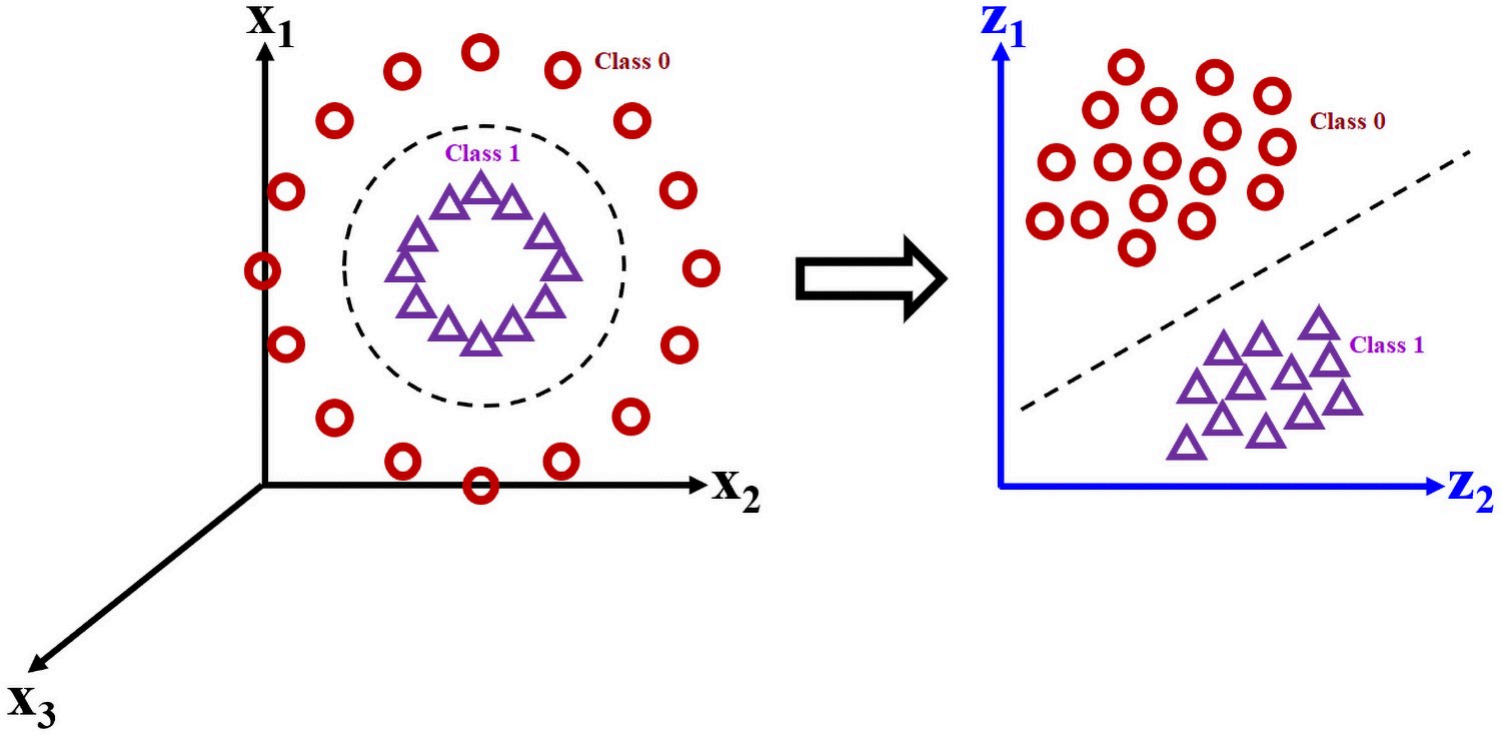
Visualization of a classification problem with a non-linear separation boundary between the two classes. *(Left)* The raw feature-space spanned by raw variables ***x***_1_ and ***x***_2_ renders linear separation impossible. *(Right)* A desired latent feature space which optimally separates the two classes. The goal of the DL model is to learn its coordinates, ***z***_1_ and ***z***_2_.

Consider a typical binary classification problem, which has a total of *C* = 2 classes (Fig 7). In many cases, due to non-linear interactions between the raw variables, the classes cannot simply be separated using a linear hyperplane (or separating boundary). Therefore, linear classifiers such as traditional Support Vector Machine (SVM) [37] will often generalize poorly for new samples (i.e. have a low test-set accuracy).

Although this non-linear classification problem can be solved with a sufficient number of iterations using One-Versus-One (OVO) and One-Versus-Rest (OVR) SVM variants [11], finding a latent space which optimally separates the two classes would arguably be a more prudent strategy. Instead of assigning the functional form of the latent space *a priori* by using kernels, we instead propose the use of a Deep Learning model which optimizes the following Eq 4, expressed in words:

$$\begin{array}{c} \begin{array}{cc} \underset{\text{parameters}}{min}\;\;\;\;\;- & \left\{\text{separating}\;\text{margins}\}\;\;+\;\;\{\text{misclassification}\;\text{rate}\right\}\\ \text{subject}\;\text{to}\;\;\;\; & \text{separating}\;\text{margins}\;\;<\;\;\infty \end{array} \end{array}$$ (4)

Note that this objective function is expressed as a *minimization*, which is standard in Machine Learning literature. Minimizing the negative margins is equivalent to maximizing the margins, under the constraint that the margins remain finite. If this constraint did not exist, then one could arbitrarily choose a latent space where the two classes shown in the right figure of Fig 7 are pushed infinitely far away from each other.

**Fig 8:**
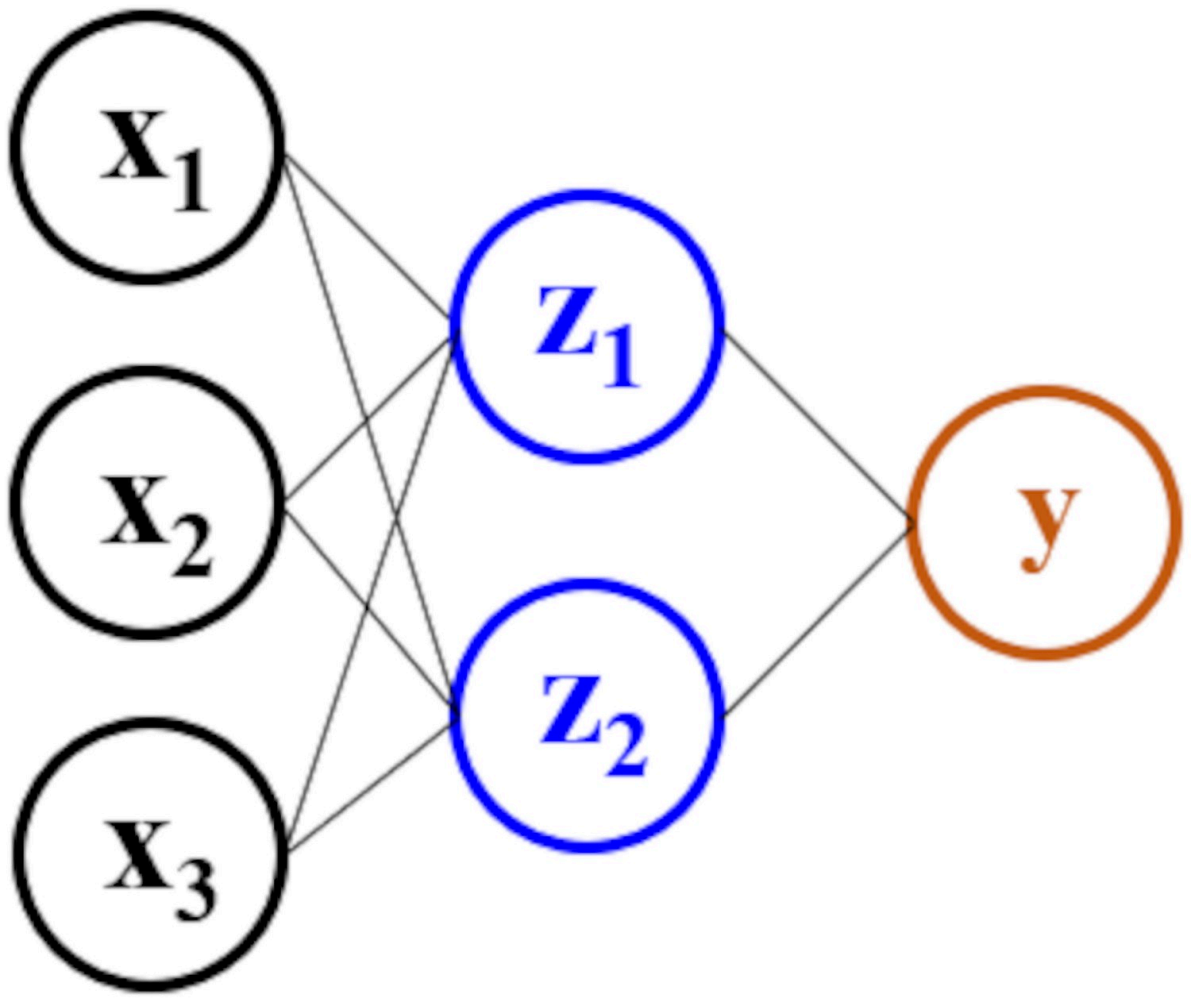
A two-layer neural network with a hinge-loss-like objective function.

Eq 4 is inspired by the *hinge loss* (Eq 2), the original objective function in the SVM paper by [37]. In Eq 2, the term ***w*** refers to the weights or parameters of the SVM model, and the term ∥***w***∥_2_ is exactly the inverse of the separating margins. Therefore, the inclusion of the term ∥***w***∥_2_ within the minimization is equivalent to sustaining the finite separating margin constraint outlined in Eq 4. The last summation term represents the total number of misclassified samples, and when visualized on a 2-dimensional plot gives a hinge-like shape (hence its name). The concept of maximizing accuracy and separating margins simultaneously can be extended to DL models. For instance, consider a two-layer neural network as shown in Fig 8.

The square-bracket superscript ^[*L*]^ refers to the *L*^*th*^ hidden layer, as conforming to the neural network nomenclature in [25]. The input layer ***X*** can be considered the first layer in the network, ***Z***^[0]^. Since the interclass separation occurs between layers ***Z***^[1]^ and ***y***, the hinge loss in Eq 2 can be re-written in terms of the neurons and parameters in said layers. Since ***y*** = ***A***^[2]^(***w***^[2]^ ***z***^[1]^ + ***b***^[2]^) and ***z***^[1]^ = ***A***^[1]^(***w***^[1]^ ***x*** + ***b***^[1]^), the hinge-loss with respect to this network is:

$$\begin{array}{c} \begin{array}{cc} \underset{w^{\left[1\right]},\;w^{\left[2\right]}}{min}\frac{1}{2}{\left|\left|w^{\left[2\right]}\right|\right|}_{2} & +\sum_{i=1}^{N}max\{0,1-A^{\left[2\right]}(w^{[2]⊤}A^{\left[1\right]}\left(w^{[1]⊤}x+b^{\left[1\right]}\right)+b^{\left[2\right]})\cdot \\ & w^{[2]⊤}\cdot A^{\left[1\right]}\left(w^{[1]⊤}x+b^{\left[1\right]}\right)\} \end{array} \end{array}$$ (5)

**Fig 9:**
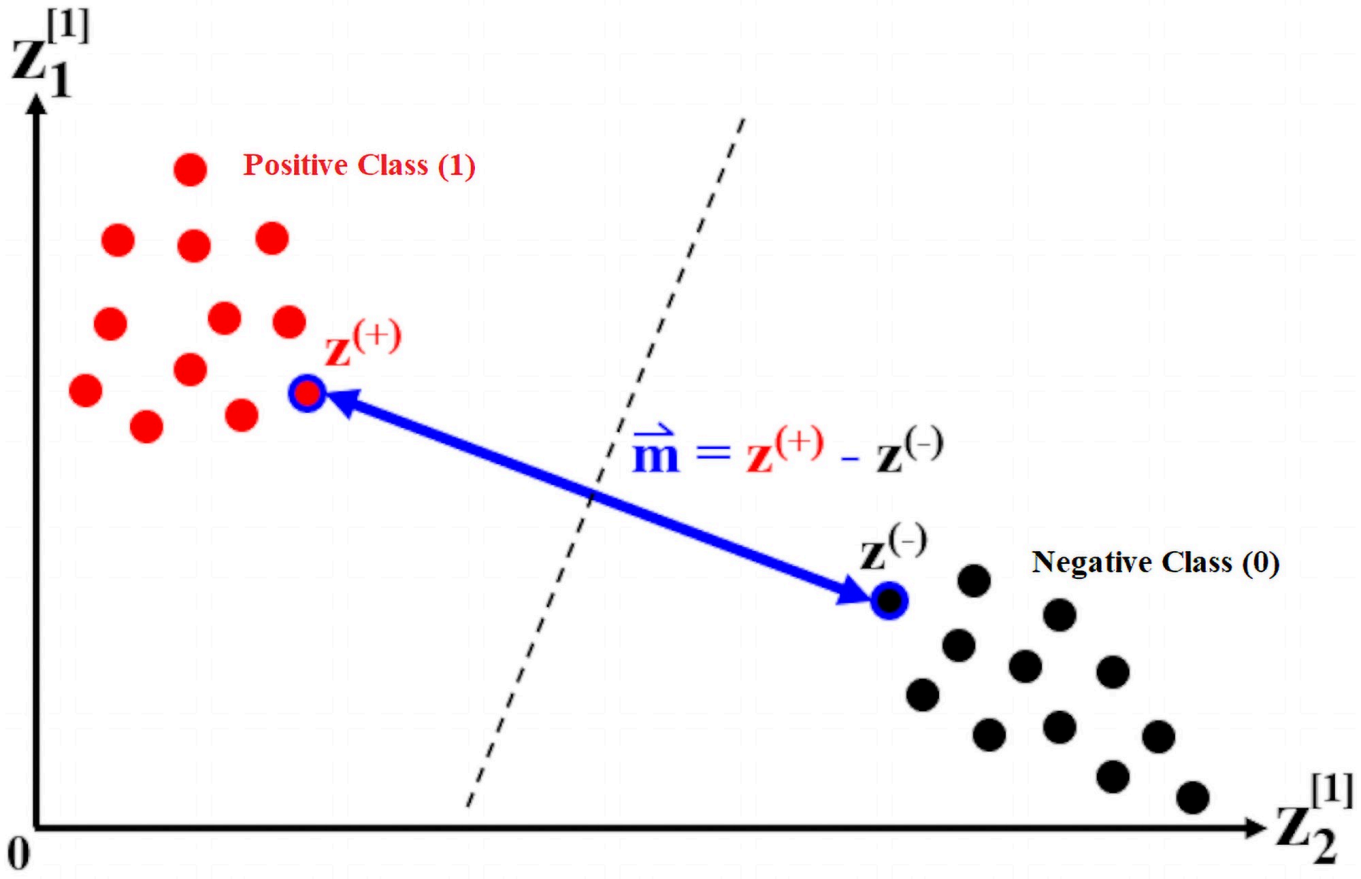
Feature selection based on direction of optimal separation.

Once this neural network has been sufficiently trained (i.e. value of its loss function has decreased past an acceptable threshold), the combination of raw inputs with the largest impact on the outcome can now be identified. Here, we propose a *feature extraction* method which takes direct advantage of the optimal separating latent space discussed previously. The concept behind this method is illustrated in the following Fig 9.

In order to easily visualize this concept, we illustrate it using the special case of a 2-neuron first hidden layer, and point out that the idea applies for any number of neurons *k*^[1]^. We consider the first hidden layer because it is optimally-separated due to Eq 5. It is also located right after the input layer in the neural network architecture, which means that it is directly affected by the magnitudes of both weights ***W***^[1]^ and input values ***X***. In this 2-dimensional hidden layer, $z_{1}^{\left[1\right]}$ and $z_{2}^{\left[1\right]}$ represent the two neurons. The positive and negative classes are separated from one another by a separating margin, which is equal to the Euclidean distance between the two closest points from each opposing class. Moreover, the directional vector of this separating margin can be characterized by $\vec{m}$, which is simply the subtraction of the coordinates between the two closest opposing samples, ***z***^(+)^ (positive class) and ***z***^(−)^ (negative class). Intuitively, $\vec{m}$ represents the direction which best classifies the two opposing classes, as opposed to any other direction found in the latent space spanned by $z_{1}^{\left[1\right]}$ and $z_{2}^{\left[1\right]}$. This property can be realized by projecting all data samples onto the vector $\vec{m}$, observing the class separation on this 1-dimensional line, then repeating the exercise for any other directional vector and comparing the resulting class separations. The elements of $\vec{m}$ can be expressed in the general case (for any value of *k*^[1]^) as:

$$\begin{array}{c} \vec{m}=[\begin{array}{cccc} m_{1} & m_{2} & \cdots & m_{k^{\left[1\right]}} \end{array}] \end{array}$$ (6)

Since the direction of $\vec{m}$ is characterized by its largest elements, we specify a number *top*_*n*_ then extract the same number of top elements (by magnitude). These can top elements can then be collected and expressed as:

$$\begin{array}{c} {\vec{m}}_{top}=[\begin{array}{ccc} m_{top_{1}} & \cdots & m_{top_{n}} \end{array}] \end{array}$$ (7)

Each of the top elements corresponds to a neuron in the first hidden layer, and hence can be expressed as an activated affine combination of input variables. For instance, if we consider the neuron corresponding to the largest element $m_{top_{1}}$, then the following equation holds for any data sample (*i*):

$$\begin{array}{c} z_{top_{1}}^{\left[1](i\right)}=A^{\left[1\right]}(w_{11}^{\left[1\right]}x_{1}^{\left(i\right)}+w_{12}^{\left[1\right]}x_{2}^{\left(i\right)}+\cdots +w_{1d}^{\left[1\right]}x_{d}^{\left(i\right)}+b_{top_{1}}) \end{array}$$ (8)

From the previous Eq 8, the *top*_*w*_ largest weights (by magnitude) can then be selected from the weight array $\left[\begin{array}{ccc} w_{11} & \cdots & w_{1d} \end{array}\right]$ as:

$$\begin{array}{c} w_{top}=[\begin{array}{ccc} w_{top_{w1}} & \cdots & w_{top_{w}} \end{array}] \end{array}$$ (9)

**Fig 10:**
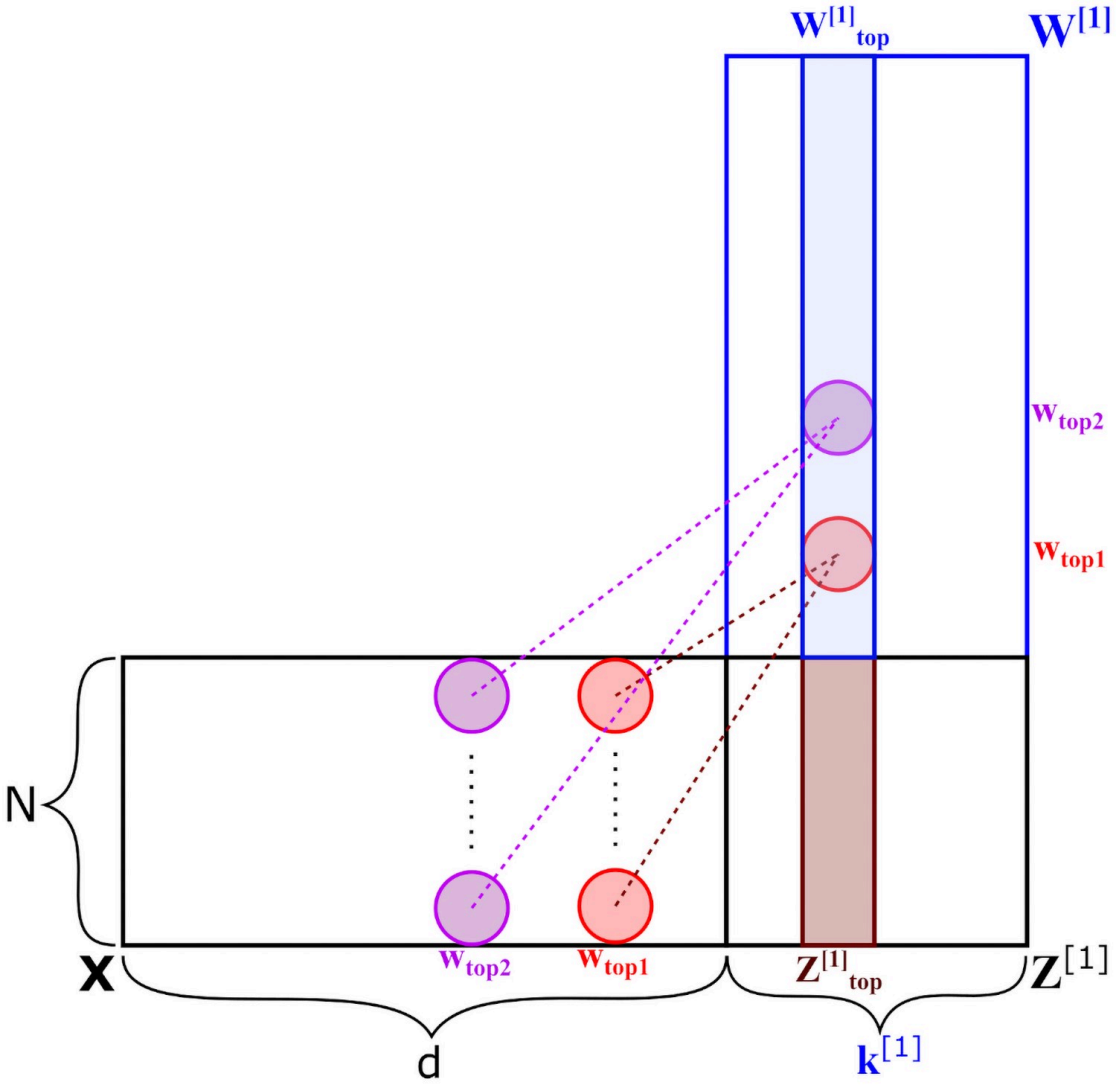
Selection of relevant input variables, by reverse-engineering matrix multiplication.

The input variables multiplied to these top weights, $\left[\begin{array}{ccc} x_{top_{w1}} & \cdots & x_{top_{w}} \end{array}\right]$, are therefore the input combinations with most impact on the model output. The rationale behind this weight selection procedure can be best visualized using the basic concepts of matrix multiplication, and is clarified using a simple example shown in Fig 10.

In this example, the first layer values ***Z***^[1]^ are a result of matrix multiplication between the input matrix ***X*** and the first hidden layer weight matrix ***W***^[1]^. Suppose the direction of separation $\vec{m}$ has its largest vector element located at position *top*, and we only consider this top element (i.e. *top*_*n*_ = 1). The values in column $Z_{top}^{\left[1\right]}$ are a direct consequence of matrix multiplication between all *d* columns in ***X*** and all elements in the column $W_{top}^{\left[1\right]}$. Therefore, if we now identify the *top*_*w*_ = 2 largest elements in this column, their positions $w_{top_{1}}$ and $w_{top_{2}}$ correspond to the two most impactful input variables within the set $\left[\begin{array}{cccc} x_{1} & x_{2} & \cdots & x_{d} \end{array}\right]$.

The entire feature extraction procedure can be summarized as follows. First, train a binary classifier with the loss objective specified in Eq 5, until the loss value falls below an acceptable threshold. Repeat model training over a sufficiently-large number of experiments, such that the results from each random initialization can be later aggregated. Then, perform the following for each model training experiment:

1. Calculate the Euclidean distance matrix between all pairwise samples in the two binary classes. Identify the two opposing-class samples ***z***^(+)^ and ***z***^(−)^ that span the separating margin as the pair with the shortest distance.
2. Calculate $\vec{m}$, the direction of separation, by substracting either the coordinates of ***z***^(−)^ from those of ***z***^(+)^, or vice versa.
3. Calculate the absolute values of all elements in $\vec{m}$, then identify the *top*_*n*_ largest elements based on these absolute values.
4. For each top neuron $z_{top_{j}}$, identify the *top*_*w*_ largest weights in the activated affine combination $z_{top_{j}}^{\left[1](i\right)}=A^{\left[1\right]}(w_{11}^{\left[1\right]}x_{1}^{\left(i\right)}+w_{12}^{\left[1\right]}x_{2}^{\left(i\right)}+\cdots +w_{1d}^{\left[1\right]}x_{d}^{\left(i\right)}+b_{top_{1}})$. The combination of input variables $\left[\begin{array}{ccc} x_{top_{w1}} & \cdots & x_{top_{w}} \end{array}\right]$ multiplied to these top weights are therefore the most impactful features.

In order to reduce variance caused by random initializations of the DL model, this process should be repeated over a large number of experiments. In each experiment, a DL model should be initialized from scratch and trained, and the extracted weights from each experiment should be recorded. Once all experiments are complete, the most impactful weights can then be ranked by majority vote across all experiments. Note that in Step 4 of the previous procedure, the *top*_*w*_ largest weights can be identified based on absolute values, if the goal is to simply extract a set of the most relevant features. However, if a distinction is required between positively and negatively-correlated weights, then the weight ranking should be performed in terms of their unmodified values.

### Summary of data analysis steps

**Fig 11:**
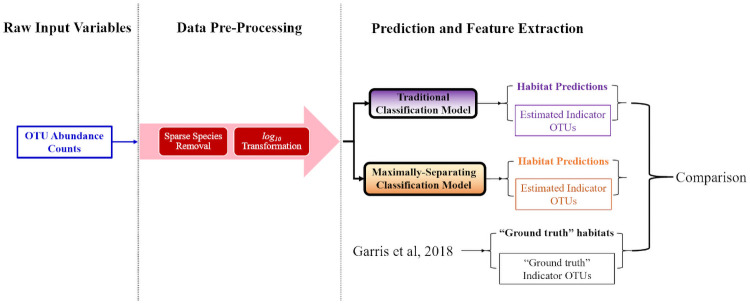
The overall data analysis workflow applied to the Mount Polley case study.

The following Fig 11 is a visualization of the data analysis procedure detailed in the previous sections, which transforms raw input data into a set of predictions as well as relevant extracted features.

The pre-processed data is used to train two classification models. The first is a traditional Deep Learning classifier, with the expected *softmax* loss function. The second is the proposed *optimally-separating* Deep Learning classifier, which uses Eq 5 as its loss function in order to improve clarification between the data classes. Two important results from each model—the class (habitat) predictions and extracted features (indicator species)—are compared and contrasted against the results cited in [34].

### Deep Learning model implementation

The Deep Learning models were implemented by code written in *iPython 3.7* Jupyter notebooks, using the *PyTorch* package. *PyTorch* was preferred over *Tensorflow* for two reasons. First, the customization of the activation functions and order of computations is less cumbersome in *PyTorch*. Secondly, the proposed optimally-separating cost function in Eq 5 requires an optimization to be performed directly on the neural network parameters. These parameters can be directly obtained in *PyTorch*, by recursively storing the parameters calculated during each epoch then recalling them. In *Tensorflow* and *keras*, these parameters are much more difficult to access and modify.

We will now describe in detail the architecture of each ANN constructed, which can be generalized using a common set of rules. The layers of each ANN can be generalized as follows:

1. **Layer 0**: The input layer, with number of dimensions equal to the number of raw features (in this case, number of species).
2. **Layers 1 ∼ (*L* − 1)**: The hidden layers, with number of neurons in each layer stored in an array.
3. **Layer *L***: The output layer, with *C* total neurons and a *softmax* activation for classification.

The neural network therefore has *L* + 1 total layers, *L* layers if the input layer is ignored, and *L* − 1 layers if only the hidden layers are counted (i.e. excluding both input and output layers). The sequence of computations in the *l*^*th*^ hidden layer, applied to each data sample, can be described as follows:

1. **Affine function**: ***w***^[*l*]⊤^***z***^[*l*−1]^ + ***b***^[*l*]^
2. **Activation function**: ***z***^[*l*]^ = ***A***^[*l*]^(***w***^[*l*]⊤^ ***z***^[*l*−1]^ + ***b***^[*l*]^)

The affine function is performed on the neuron values obtained from the last layer, ***z***^[*l*−1]^. The neuron values of the current layer, ***z***^[*l*]^, is the value of the affine function transformed by the currently desired activation, ***A***^[*l*]^. This activation is chosen as *ReLU* for all neural networks used in this study. Finally, the output layer (layer *L*) of each ANN obeys the following sequence of computations:

1. **Affine function**: ***w***^[*l*]⊤^***z***^[*l*−1]^ + ***b***^[*l*]^
2. **Activation function**: ***A***^[*l*]^(***w***^[*l*]⊤^***z***^[*l*−1]^ + ***b***^[*l*]^)
3. ***Softmax*****transformation**: ***z***^[*L*]^ = *softmax*(***A***^[*L*]^(***w***^[*L*]⊤^ ***z***^[*L*−1]^ + ***b***^[*L*]^))

At the last (*L*^*th*^) layer, *C* neurons are present, with each neuron value representing a *logit*. These can be considered unscaled *log*-likelihoods of the data sample belonging to each class. More specifically, *logit* 1 represents the log-likelihood of the sample belonging to Class 1, going all the way up to *logit* *C* with a similar interpretation. The final *softmax* function transforms all these *logits* into proper probabilities that sum up to 1. Hence, the final array outputted by the *softmax* contains *C* elements, with each element representing the 0 ∼ 1 probability that it belongs to class *c* ∈ *C*.

In order to achieve a rational compromise between *underfitting* and *overfitting*, each ANN is set to train for a maximum of 1000 epochs, with two overfitting mitigation strategies employed:

1. **Regularization**: An *ℓ*_2_-regularizer is introduced in each layer, which adds the term *α*∥***w***^[*l*]^∥_2_ to the cost function (Eq 5) for the weights in all layers.
2. **Early-stopping**: The loss value from Eq 5 is calculated and stored for every epoch. If the loss values have not changed by more than *ES*_*thresh*_ over the last *n*_*ES*_ epochs, then the training terminates prematurely even if the maximum number of epochs has not been reached.

The regularizer coefficient *α* is typically set to 0.01. The number of epochs for early-stopping *n*_*ES*_ is set to 5, and the early-stopping threshold value *ES*_*thresh*_ is set to 10^−3^. These values may differ slightly for each ANN in order to maintain the underfitting-overfitting balance.

**Fig 12:**
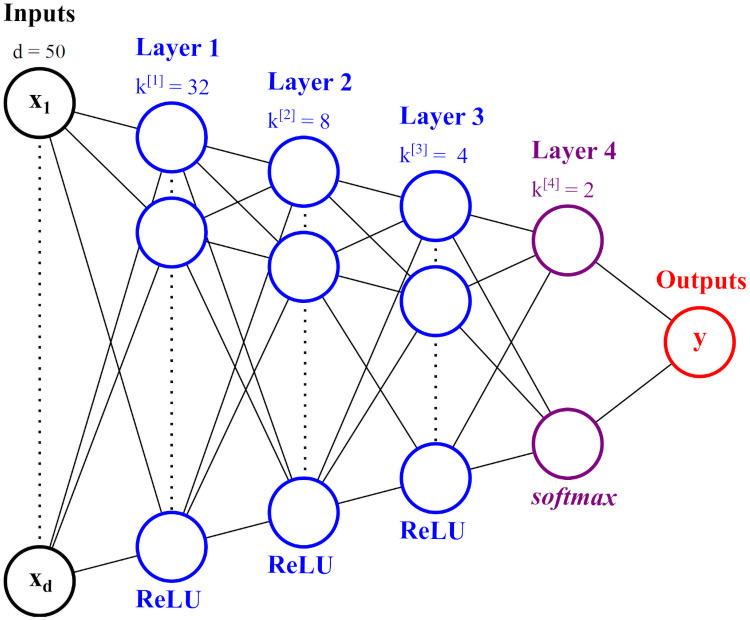
The ANN neuron architecture used in the Mount Polley case study.

The ANN used for indicator identification in the environmental case study can be visualized in the following Fig 12. The number of raw variables in the input layer is *d* = 50. The number of neurons in the first hidden layer of the ANN is usually determined by systematic search methods such as [40, 41], or simply specified as a value close to the number of raw variables *d*. Therefore, in this particular case study, we have chosen 32 as the number of neurons in first hidden layer. The second hidden layer uses 8 neurons; this compression serves as a “feature mapping” which extracts only the most relevant latent combinations for classification. Similarly, the third hidden layer uses 4 neurons. Finally, the last hidden layer uses 2 neurons, since this number must be equal to the total number of classes *C* = 2 in order for the *softmax* activation to make sense. Each hidden layer uses a *ReLU* activation, except for the last layer which uses the *softmax* activation required for classification.

## Results

The efficacy of the optimally-separating feature extractor is first demonstrated on the well-known benchmark dataset, the Fisher *Iris* [23]. Two Deep Learning models were built and trained with identical architectures. The only difference in these models was the loss function: one used a traditional *softmax* loss whereas the other used the proposed optimally-separating loss. The results show that the optimally-separating model produces much larger separating margins between the binary classes, whilst still achieving perfect test accuracy when compared to the traditional. It is also capable of identifying the same relevant features in the *Iris* problem as those expected from domain knowledge.

The second portion of the results will demonstrate the optimally-separating feature when applied to the environmental monitoring case study. We will show that the keystone microorganisms identified using our proposed method are similar compared to the ones found by [34], in terms of taxonomic composition.

### Proof of concept: The Fisher *Iris* dataset

#### Problem statement

The Fisher *Iris* [23] dataset contains *N* = 150 total samples, with *d* = 4 features:

- ***x***_1_: Sepal length (cm)
- ***x***_2_: Sepal width (cm)
- ***x***_3_: Petal length (cm)
- ***x***_4_: Petal width (cm)

The three types of *Iris* flowers or outcomes, split evenly with 50 samples each, are:

- *y*_1_: *Iris Setosa*
- *y*_2_: *Iris Versicolor*
- *y*_3_: *Iris Virginica*

Domain knowledge states that the flower types are decided according to the last two features, ***x***_3_ (Petal length) and ***x***_4_ (Petal width), whereas the first two features (sepal length and width) have a much less noticeable impact. In order to formulate this problem as a binary classification task, we only consider the first *N* = 100 samples of the dataset, which includes the two outcome classes of *Iris Setosa* (Class 0) and *Iris Versicolor* (Class 1).

**Fig 13:**
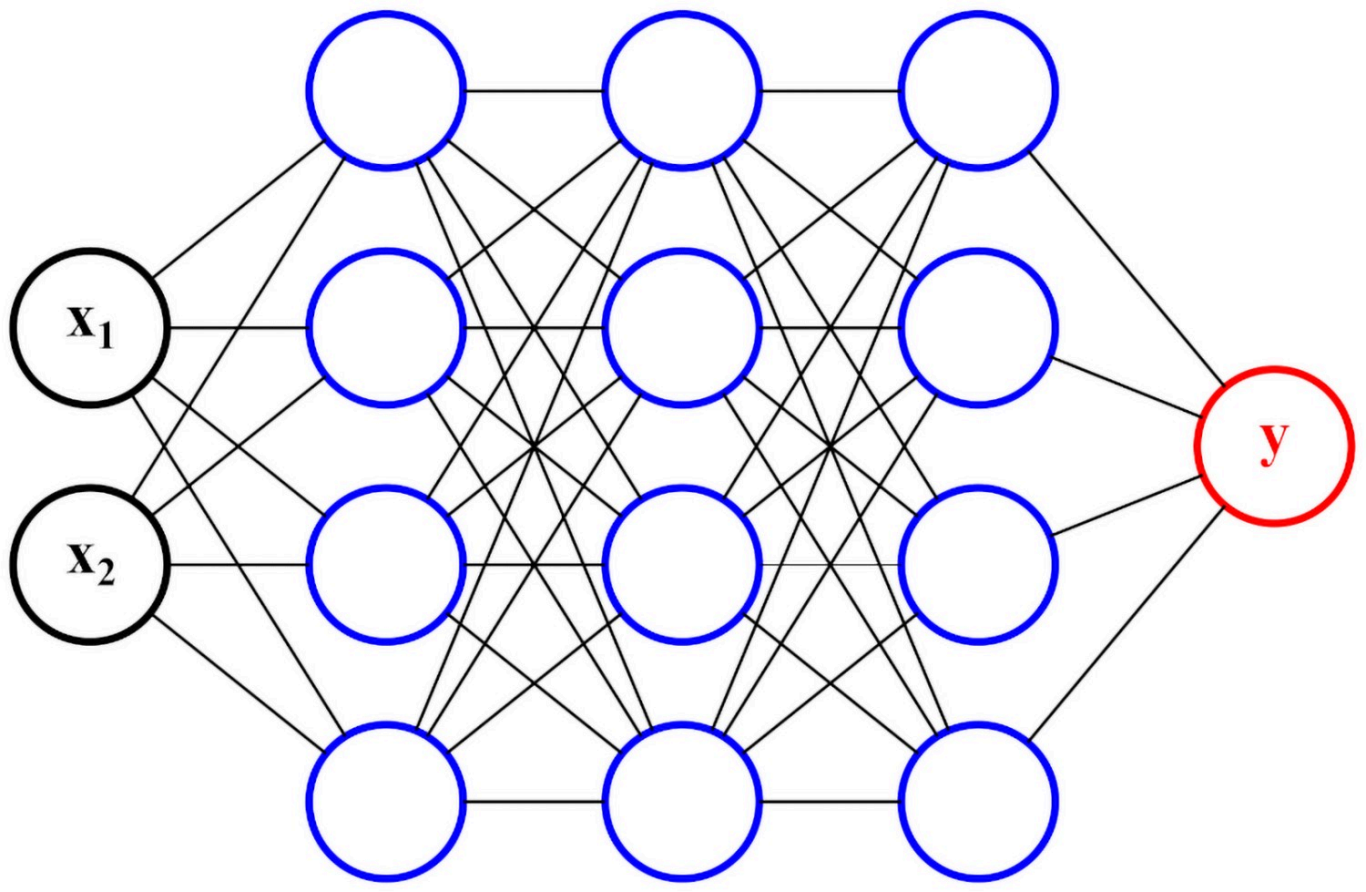
The 4-layer ANN architecture used to classify the Fisher *Iris* dataset.

For this proof-of-concept, two ANNs are trained—a traditional ANN with the typical *softmax* loss function, and an optimally-separating ANN with the loss function specified in Eq 5. Both ANNs are constructed with the architecture shown in Fig 13, and hyperparameters detailed in Table 1.

**Table 1:** *Iris* ANN classifier details.

Hyperparameter	Value/Type
Activation in each layer (except last)	*ReLU*
Train:Test ratio	80: 20
Early-Stopping criterion	Average loss over past 5 iterations differs by no more than 1%
Total epochs per experiment	1000
Total experiments	10000

Note that the training and testing samples, while being randomly selected, is performed such that the ratio of positive to negative class samples is preserved to be the same as in the overall dataset. Moreover, each ANN model is trained over a large number of experiments, since the ANN parameters may initialize in poor locations that converge to high local minima far away from the true global minimum. These result in different top weights, and consequently, different features being extracted. The results are aggregated across all experiments. For each experiment, random training and testing sets are selected with a 80: 20 train:test split ratio.

The results obtained from these two types of loss functions will be compared against one another using the following metrics:

- Model accuracy on the training and testing sets.
- Separating margin between the two classes, i.e. Euclidean (*ℓ*_2_) distance between the two closest opposing samples.

After each of the 10000 ANN models is trained for each case, we first compare the training and testing accuracies of each ANN model, as shown in Table 2.

**Table 2:** ANN training and testing accuracies on the *Iris* dataset.

Model	Average Training Acc. (%)	Average Testing Acc. (%)
Traditional	100	100
Optimally-Separating	100	100

Since the *Iris* benchmark is a fairly straightforward classification problem, it is no surprise that both ANN models are able to achieve perfect training and testing accuracies, especially when the samples have been reduced to a binary set.

Now that the model generalization accuracy has been ascertained, we compare the inter-class separation distances in each hidden layer.

**Fig 14:**
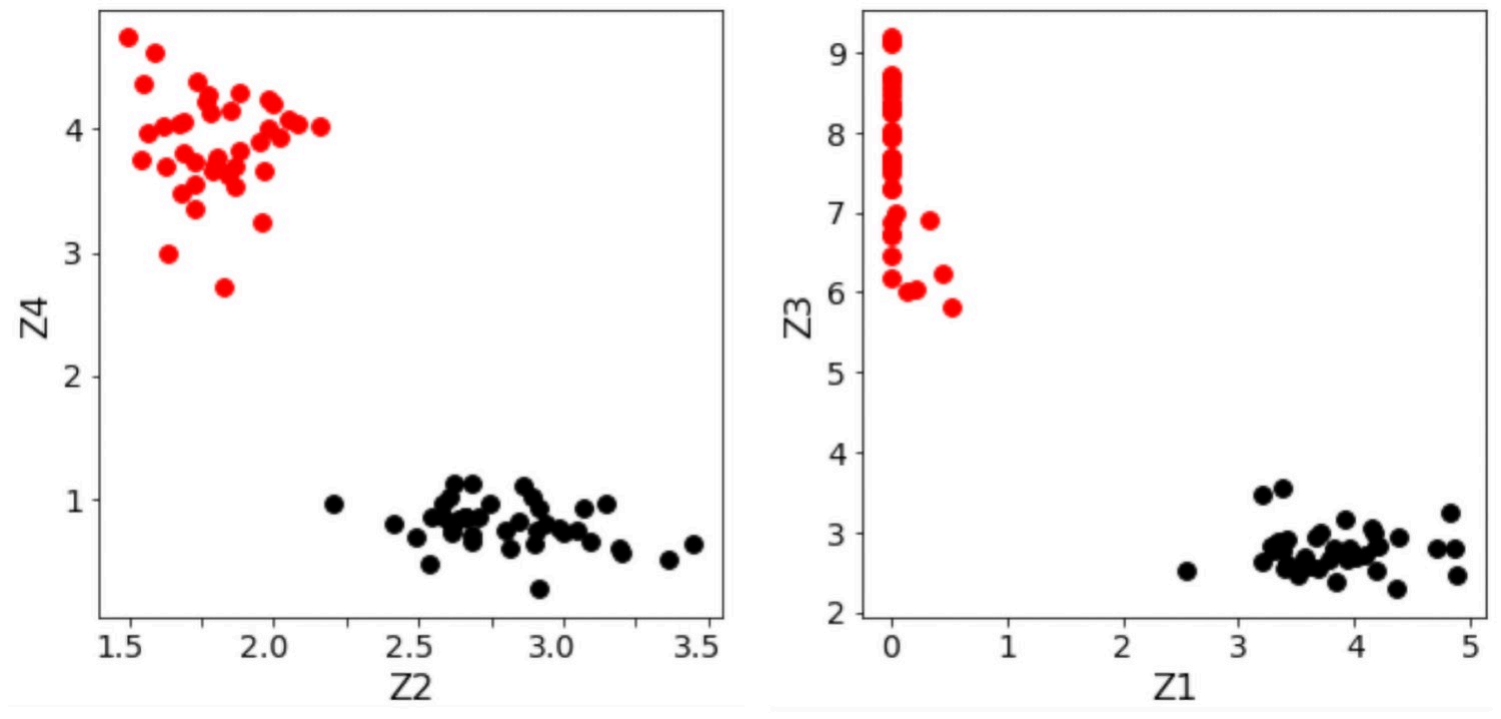
Visualization of data separation within the first ANN hidden layer. *(Left)* Inter-class separation distance in the traditional ANN, between Class 2 (red) samples and Class 1 (black) samples. *(Right)* Inter-class separation distance in the optimally-separating ANN.

The results in Table 3 show that the positive and negative class clusters in the optimally-separating ANN have been pushed much farther apart, than in the traditional ANN counterpart. This indicates that the hinge-like loss in Eq 5 is working as intended. However, both ANNs achieve 100% accuracy due to the fact that the two classes are easily separated, as shown in Fig 14.

**Table 3:** Comparison of separating margins between traditional and optimally-separating ANNs, for the *Iris* classification problem.

Sep. Margin	Traditional			Max. Separating		
Hidden Layer	1	2	3	1	2	3
**Avg**	1.78	1.87	2.04	6.28	23.27	107.7
**Max**	2.14	2.37	2.63	13.49	84.24	506.9
**Min**	1.33	1.36	1.33	1.70	3.45	40.9

Finally, we now analyze the feature extraction capabilities of the proposed, optimally-separating ANN. Following the weight-ranking procedure outlined in the previous *Proposed Deep Optimal Feature Extractor* section, we selected *top*_*n*_ = 2 largest vector elements, then the *top*_*w*_ = 2 largest input weights for each top vector element. The input weights were ranked using their absolute values (rather than their unmodified values), due to no need of differentiation between positively and negatively-correlated weights. The final input variable rankings, i.e. fraction of experiments in which each input variable $\left[\begin{array}{cccc} x_{1} & x_{2} & x_{3} & x_{4} \end{array}\right]$ was identified as having the top weights, are reported in Table 4.

**Table 4:** Feature-ranking of the *Iris* dataset, according to our proposed optimally-separating ANN.

Input Variable	Frequency (%)
Sepal Length (***x***_1_)	0.0
Sepal Width (***x***_2_)	0.0
Petal Length (***x***_3_)	54.2
Petal Width (***x***_4_)	47.8

The results indicate that the combination of *Petal Length* (***x***_3_) and *Petal Width* (***x***_4_) input features dominate in terms of deciding the final binary outcome. This agrees with the aforementioned domain knowledge of this well-known dataset. Note that these ranking percentages may change significantly, since we only set a threshold of 10000 experiments due to the limitations of our computing power. However, if a sufficiently large number of experiments could be conducted (ex. 100000 or more), then a more reliable and consistent ranking list could be obtained.

## Indicator species case study results

The Fisher *Iris* benchmark results from the previous section indicate that our proposed feature extractor is capable of identifying the correct combination of relevant features impacting the outcome. Now we perform our proposed algorithm on the environmental monitoring dataset, and observe whether the extracted species features are similar to the indicator species identified by the authors of [34].

The first objective of the DL model is to train itself to correctly predict the habitat label of each sample to the best of its ability, given input data in the form of species abundance counts. The second objective of the DL model is to identify *indicator species* with respect to both the undisturbed and disturbed classes. By considering each class as a biocommunity, these keystone species can then be considered main actors which influence the behavior of each biocommunity as a whole. The indicators may subsequently help environmental engineers mitigate the aftermaths of similar spills in the future, by revealing potential ecological shifts in microbial communities.

Finally, after all results are presented, we will compare and contrast the indicator species identified by the Deep Learning feature extractor and the original indicators provided by [34]. The results show that although the two sets of identified indicator species show distinct differences, they also share several similarities. More importantly, the results also indicate that subsequent predictive models can be constructed with higher accuracy when using the new indicator species (from our proposed method) as inputs, as opposed to using the indicators identified by [34].

### Predictive modelling results

A total of 21721 species are present in the raw dataset. However, many of these species are sparsely-occurring, meaning their abundance counts are zero across most samples. Considering the extremely small number of samples to start with (*N* = 70), we use a reasonably proportional number of features to prevent *the curse of dimensionality* as much as possible. Therefore, we have chosen to include only the top 50 species, in terms of total abundance counts across all existing samples. Using these abundance counts, we build two ANNs, both with the the architecture specified in Fig 12. One of the ANNs is an optimally-separating one, which uses the loss function prescribed in Eq 5. The other ANN is, of course, a traditional one with a straightforward *softmax* loss. Both traditional and optimally-separating ANNs were trained and tested on the Mount Polley dataset. A comparison of their accuracies (average, maximum, minimum and standard deviations) across 5000 runs (for each case) can be observed in the following Table 5.

**Table 5:** Testing-set accuracy comparison between traditional and optimally-separating ANNs.

Test Acc. (%)	Traditional	Optimally Separating
**Avg**	98.6	98.7
**Max**	100.0	100.0
**Min**	84.6	84.6
**Std**	2.1	2.1

**Fig 15:**
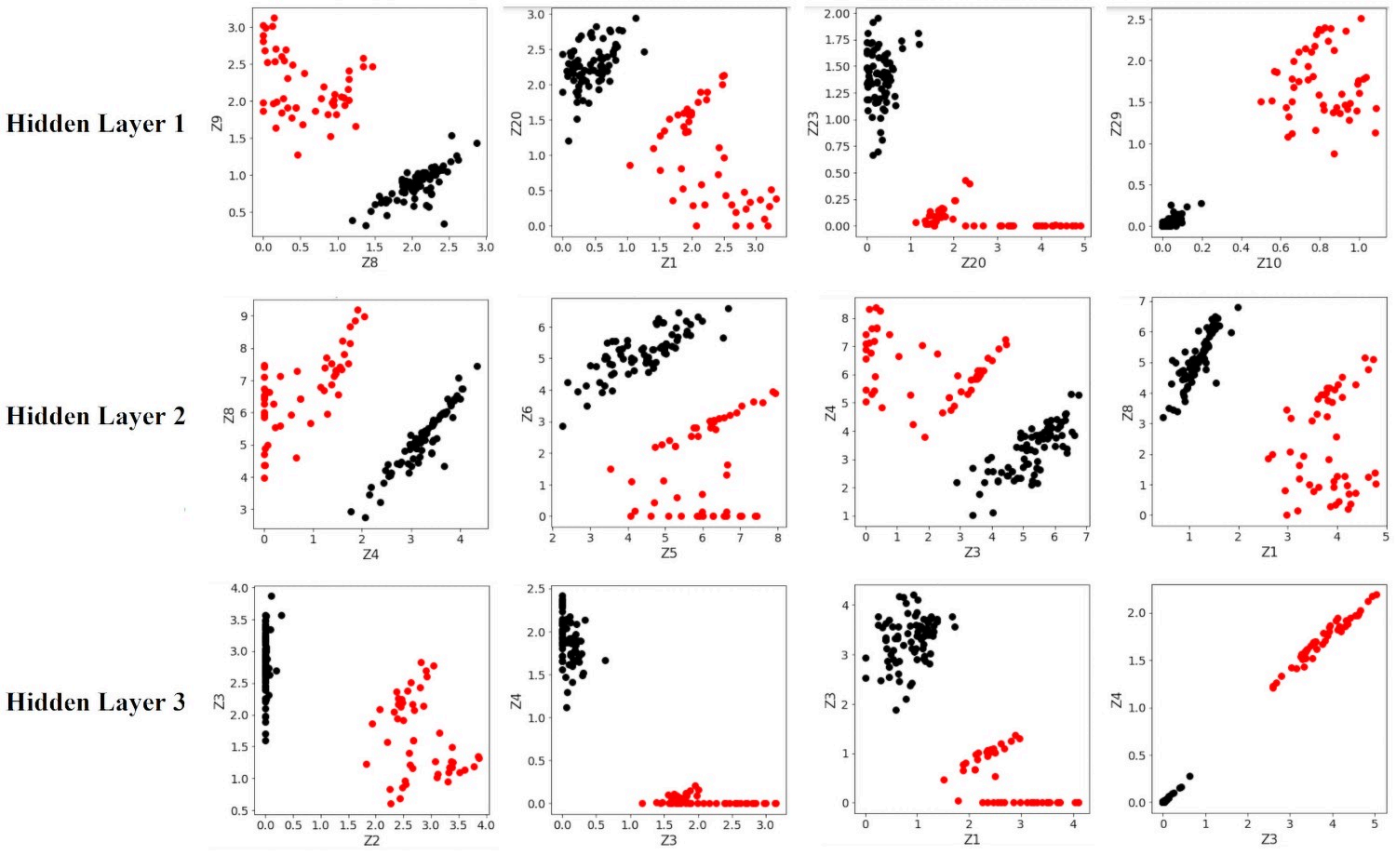
Inter-class separations in the traditional ANN. *Black* samples belong to the undisturbed class. *Red* samples belong to the disturbed class. Only the first 4 out of the total 5000 runs are shown.

**Fig 16:**
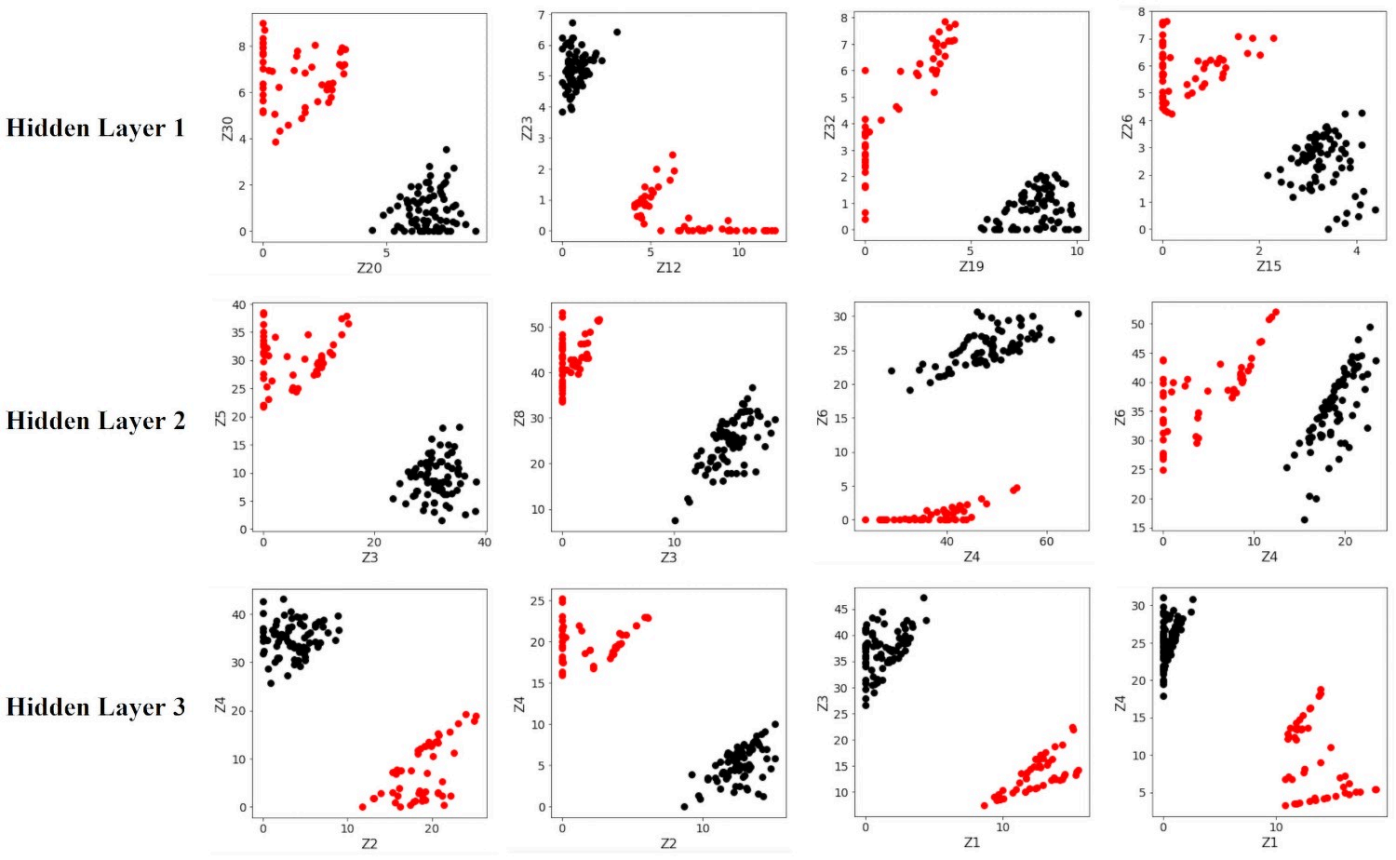
Inter-class separations in the optimally-separating ANN. The separations are noticeably larger than those in Fig 15. *Black* samples belong to the undisturbed class. *Red* samples belong to the disturbed class. Only the first 4 out of the total 5000 runs are shown.

Note that the test accuracies are essentially the same across the two types of ANN models. The main reason behind this phenomenon can be observed in the following Figs 15 and 16, which shows two-dimensional plots of the largest separation margins within the layers of each ANN, for the first 4 experiments. Notice that while the separating margins in the traditional ANN (Fig 15) are significantly smaller (in terms of Euclidean distance) compared to those in the optimally-separating ANN (Fig 16), in both cases perfect separation of the binary classes are achieved. Therefore, the increased separating margin does not result in a visible increase in prediction accuracy, as it is expected to do so for cases where the traditional ANN would be unable to cleanly separate the classes.

The exact Euclidean distances of the separating margins, averaged across all 5000 runs, are tabulated in the following Table 6. Notice that the increases in margins afforded by the optimally-separating ANN range between 3 to 10 times on average.

**Table 6:** Comparison of separating margins between traditional and optimally-separating ANNs.

Sep. Margin	Traditional			Optimally Separating		
Hidden Layer	1	2	3	1	2	3
**Avg**	0.88	1.50	1.37	2.77	10.67	15.21
**Max**	1.93	4.49	4.01	7.99	48.92	73.11
**Min**	0.36	0.34	0.13	1.52	4.19	6.94

After the predictive models—traditional and optimally-separating ANNs—are built and trained adequately, they are then used to extract the indicator species associated with both undisturbed and disturbed habitats. This was performed using the weight-tracing procedure outlined in the previous *“Proposed Deep Optimal Feature Extractor”* section. The set of indicators identified from the proposed method were used to train 3 types of predictive models—Random Forests, Support Vector Machines, and (traditional) Artificial Neural Networks. This procedure was then repeated using the indicators identified by [34]. The average test-set accuracies across 5000 runs, as well as their standard deviations, are tabulated in the following Table 7.

**Table 7:** Testing-set accuracy comparison between classifiers, using either all species or only indicator species as inputs. Plus-minus value represents one standard deviation.

Test Acc. (%)	50 top species (by sum)	Garris Ind. only	Estimated Ind. only
**RF**	97.9±1.4	97.36±1.6	96.62±1.7
**SVM**	98.7±1.5	97.37±1.7	98.72±1.7
**ANN**	98.7±1.6	97.41±1.8	98.71±1.8

When the top 50 species (by abundance sum) are used for training, the generalization capability of all three types of predictive models are higher compared to the case where the indicators identified by [34] are used. When the indicators estimated using the optimally-separating feature extractor are used, generalization capability increases slightly for SVM and ANN models, but decreases for the RF model. This is an interesting observation which appears opposite to the expected results between SVMs and RFs. More specifically, the SVM is a linear classifier, whereas the RF is an ensemble method which is able to map out non-linear underlying structure by sheer brute force (i.e. majority votes over a large number of experiments). Since the raw data is highly non-linear in nature, the RF is expected to perform better. Overall, the SVM and ANN test accuracies are the highest when using the estimated indicators, followed by the 50 top species, and worst when using the Garris [34] indicators. These results suggest that in terms of habitat prediction, the set of estimated indicators contain less noisy and/or irrelevant species combinations compared to both Garris [34] and the original dataset.

### Indicator species identification

The arguably more important goal of data analytics, in the context of bioinformatics, is to provide insight into the relevant features affecting each specific outcome. In this specific case study concerning the Mount Polley tailings dam breach, we identify indicator species using our proposed strategy, then compare and contrast them to the indicators identified by [34]. The results are shown in the following Table 8:

**Table 8:** Indicator species identified by our proposed feature extractor, compared to those identified by [34]. The *Frequency* column denotes the number of times each species has been identified as a top weight, divided over all 5000 experiments. The *Garris* column represents whether each species has been identified in the paper [34], and if so, which habitat it belongs to. The maximum and mean abundance counts of each species are also shown, along with the *Class* taxonomy level.

**Species ID**	Frequency (%)	Garris [34]	Mean(Natural)	Max (Natural)	Mean (Disturbed)	Max (Disturbed)	Taxonomy (Class)
**Species39**	22.00	N/A	5	28	3	35	Acidobacteria
**Species11**	10.69	N/A	26	435	12	236	Methanomicrobia
**Species1**	9.27	Disturbed	3	13	305	1310	Betaproteobacteria
**Species17**	9.21	Disturbed	2	4	35	218	Flavobacteriia
**Species53**	7.96	Natural	12	74	2	9	unclassified
**Species172**	4.62	Natural	8	36	2	7	Methanomicrobia
**Species13**	3.28	Disturbed	3	13	37	302	Gammaproteobacteria
**Species88**	3.12	Natural	6	36	2	5	Anaerolineae
**Species29**	3.08	N/A	6	56	3	9	Cyanobacteria
**Species16**	3.07	N/A	2	2	24	215	Flavobacteriia
**Species42**	1.73	Disturbed	5	28	2	3	Methanomicrobia
**Species52**	1.73	Disturbed	11	64	7	38	Flavobacteriia
**Species102**	1.70	Disturbed	4	19	28	255	Betaproteobacteria
**Species10**	1.61	N/A	8	37	6	51	Betaproteobacteria
**Species8**	1.61	Natural	8	118	9	39	Betaproteobacteria
**Species21**	1.60	Natural	9	29	4	18	Deltaproteobacteria
**Species4**	1.58	N/A	2	5	126	488	Bacilli
**Species14**	1.58	Disturbed	2	9	27	490	Gammaproteobacteria
**Species41**	1.57	Natural	13	31	3	9	Betaproteobacteria
**Species15**	1.54	Natural	16	137	3	9	Gammaproteobacteria
**Species26**	1.51	N/A	4	61	2	13	Betaproteobacteria
**Species62**	1.51	Natural	9	60	2	12	Deltaproteobacteria
**Species9**	1.50	N/A	2	4	35	129	Bacilli
**Species18**	1.49	N/A	10	97	2	5	Cyanobacteria
**Species54188**	1.49	N/A	8	53	3	12	Chloroplast

**Fig 17:**
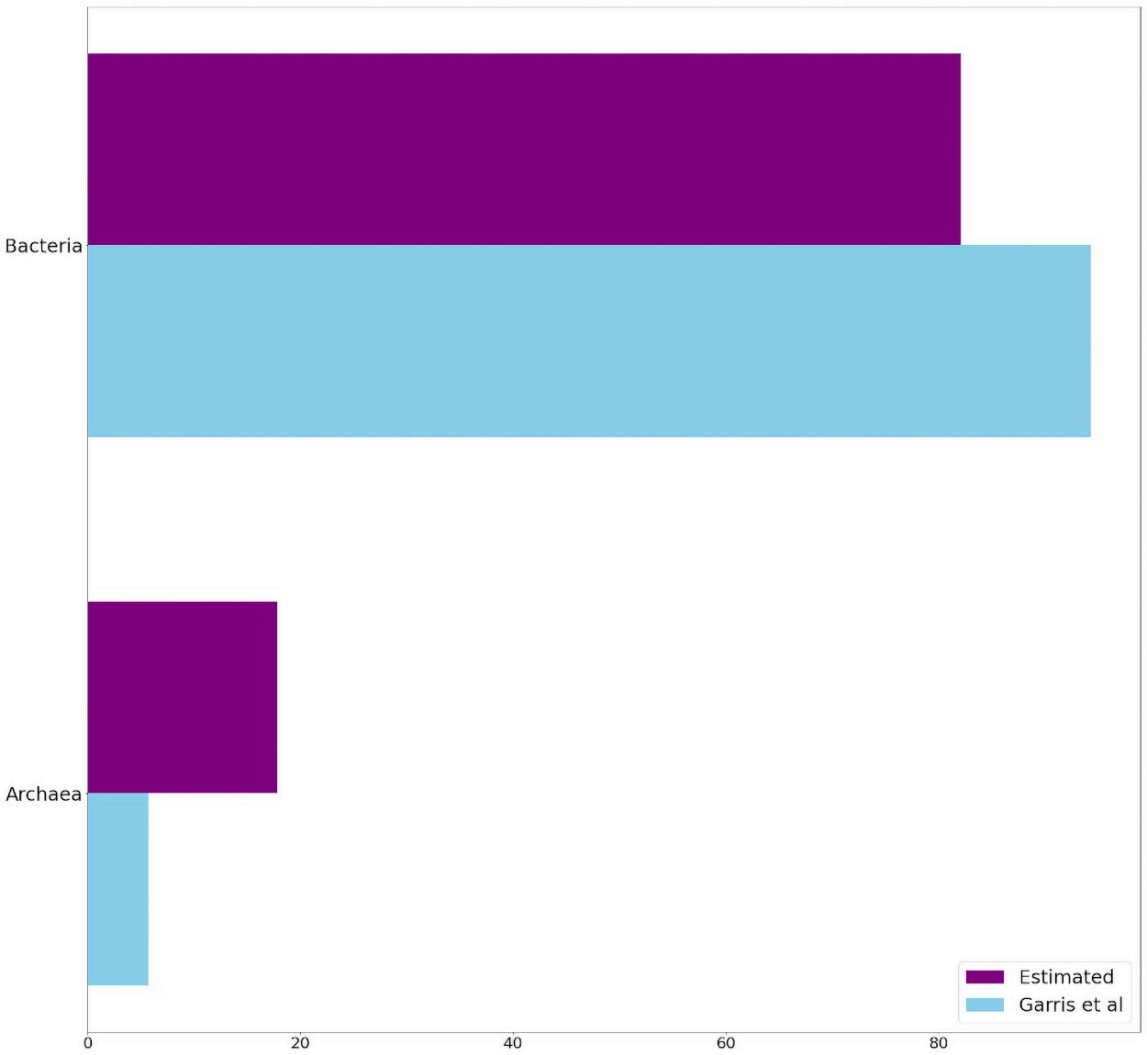
Taxonomic comparison of indicator species at the *domain* level. *Blue* bars represent the indicators identified by Garris et al [34]. *Purple* bars represent the indicators identified by our proposed feature extractor. The horizontal axis represents the percentage of indicators belonging to each species.

**Fig 18:**
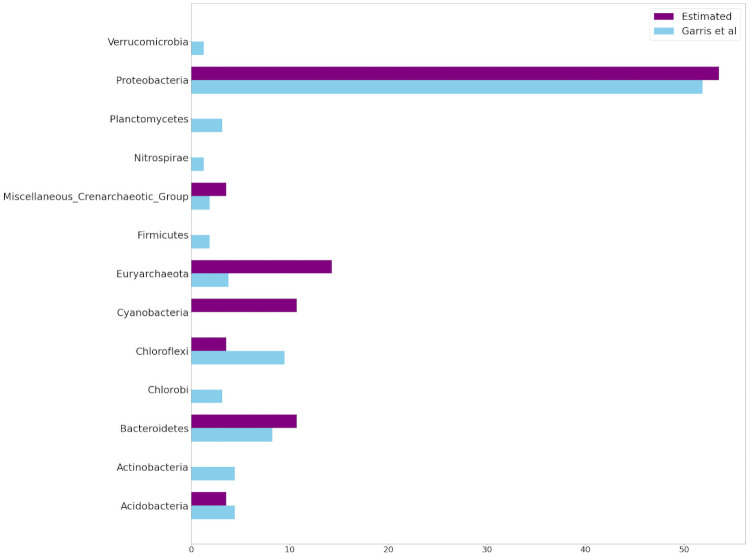
Taxonomic comparison of indicator species at the *phylum* level. *Blue* bars represent the indicators identified by Garris et al [34]. *Purple* bars represent the indicators identified by our proposed feature extractor. The horizontal axis represents the percentage of indicators belonging to each species.

**Fig 19:**
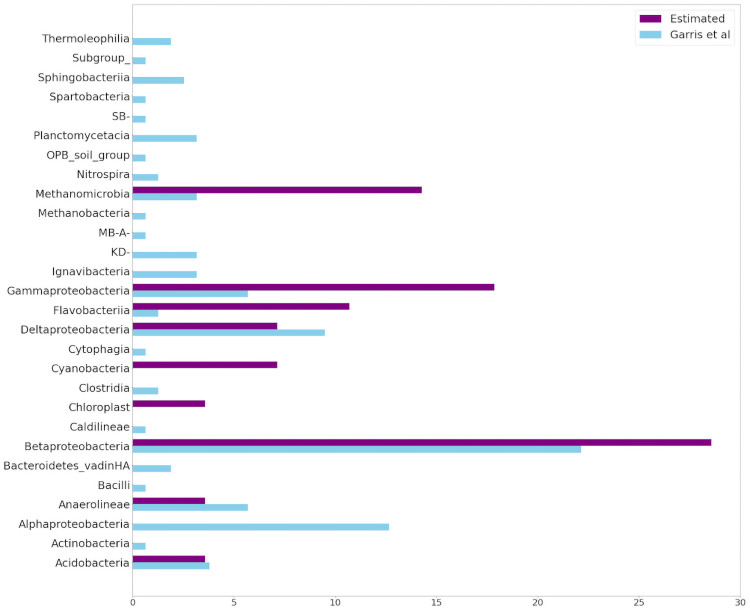
Taxonomic comparison of indicator species at the *class* level. *Blue* bars represent the indicators identified by Garris et al [34]. *Purple* bars represent the indicators identified by our proposed feature extractor. The horizontal axis represents the percentage of indicators belonging to each species.

Two important results in Table 8 are worthy of mention. First, 15 out of the 25 estimated indicators have also been previously been identified by [34]. This implies a healthy extent of overlap between the two vastly different identification methods. Moreover, *Species*1 has been identified as an indicator by both methods. This species is *lithotrophic*, (i.e. it survives by feeding off inorganic material, including solid minerals). It is abundantly present in disturbed habitats that predominantly consist of tailings, which are mineral in origin with little organic material present. In order to compare these results at a more detailed level, we report the indicator species in terms of the taxonomic levels of *Domain* (Fig 17), *Phylum* (Fig 18), and *Class* (Fig 19). The proportional total number of indicators belonging to each category within each taxonomic level can be visualized in the following Figs 17 through 19. Only the first 3 taxonomic levels were considered, as any level below *class* showed species divisions which were too fine for meaningful comparison. At the *domain* level, our proposed feature extractor identified a similar number of *Bacteria* species, but less *Archaea* species, when compared to the Garris indicators. At the *phylum* level, our method identifies *Proteobacteria* as the predominant type of indicators, which strongly agrees with the Garris results. Both methods also identify *Bacteroidetes* and *Acidobacteria* as indicator types of similar proportionality. On the other hand, our method identifies less *Euryarchaeota* and more *Chloroflexi* indicators. Finally, at the *class* level, our method identifies *Betaproteobacteria* as the predominant type of indicators, which again agrees with the Garris results. However, several indicator species have only been identified by one method alone. For example, Garris et al recognized *Cyanobacteria* and *Chloroplast* species as indicators, while none of them were recognized by our method. In contrast, our method picked up many species such as *Thermoleophilia*, *Methanobacteria*, etc., which were not recognized by Garris et al. Nevertheless, the overall results indicate a satisfactory degree of overlap between our feature extractor and that of [34].

## Conclusion

In this work, we introduce a novel feature extraction algorithm based on Deep Learning, suited specifically for binary classification problems. The algorithm uses a hinge-like loss function in place of a traditional cross-entropy loss, in order to optimally separate the two distinct classes in the discovered latent spaces. The most relevant input features are extracted by determining the vector which describes the direction of inter-class separation in the latent space, then selecting the largest elements within said vector. We first demonstrate our algorithm on the benchmark *Fisher Iris* classification problem, and show that it is capable at accurate prediction as well as identifying the correct relevant features (i.e. *sepal length* and *sepal width*). We then test our proposed strategy on an environmental monitoring dataset collected from the Mount Polley tailings breach, which includes microorganism species abundance counts from natural and disturbed habitats. The first set of results show that our proposed feature extractor identifies indicator species with similar *domain* and *phylum* taxonomic levels, when compared to those identified in the previous work [34]. The second set of results show that the indicators extracted from our proposed method lead to more accurate habitat predictions, when used in models such as Support Vector Machine or traditional Artificial Neural Network models.

Future plans to improve this work include rigorous sensitivity testing of the proposed feature extractor, as well as expanding its scope and applicability. For instance, the current formulation has only been tested on a relatively small dataset with *N* = 70 samples. It would be interesting to observe whether the same success holds for bigger datasets with thousands of samples, as Deep Learning typically trains better on larger sets [25]. Moreover, a sensitivity study which investigates performance change with respect to hyperparameters *top*_*n*_ and *top*_*w*_ (i.e. the number of top components selected in each feature extraction step) would strengthen the reliability of the algorithm. With respect to the general state of research in the feature extraction field, the main next step is to implement our method on datasets from similar disciplines such as disease (ex. COVID) diagnosis and prevention. These future case studies will produce additional results that compare and contrast the efficacy of our algorithm to well-established such as PCA-Firefly [16], and further clarify whether Deep Learning can indeed provide more intuitive and actionable feature extraction results.
